# Light pollution disrupts circadian clock gene expression in two mosquito vectors during their overwintering dormancy

**DOI:** 10.1038/s41598-024-52794-x

**Published:** 2024-01-29

**Authors:** Lydia R. Fyie, Katie M. Westby, Megan E. Meuti

**Affiliations:** 1 Department of Entomology, The Ohio State University, 2021 Coffey Rd, Columbus, OH 43210 USA; 2 Tyson Research Center, Washington University in St. Louis, 6750 Tyson Valley Road, Eureka, MO 63025 USA

**Keywords:** Animal physiology, Reverse transcription polymerase chain reaction, Entomology

## Abstract

Artificial light at night (ALAN) is an increasingly important form of environmental disturbance as it alters Light:Dark cycles that regulate daily and seasonal changes in physiology and phenology. The Northern house mosquito (*Culex pipiens*) and the tiger mosquito (*Aedes albopictus*) enter an overwintering dormancy known as diapause that is cued by short days. These two species differ in diapause strategy: *Cx. pipiens* diapause as adult females while *Ae. albopictus* enter a maternally-programmed, egg diapause. Previous studies found that ALAN inhibits diapause in both species, but the mechanism is unknown. As the circadian clock is implicated in the regulation of diapause in many insects, we examined whether exposure to ALAN altered the daily expression of core circadian cloc genes (*cycle*, *Clock*, *period*, *timeless*, *cryptochrome 1*, *cryptochrome 2*, and *Par domain protein 1*) in these two species when reared under short-day, diapause-inducing conditions. We found that exposure to ALAN altered the abundance of several clock genes in adult females of both species, but that clock gene rhythmicity was maintained for most genes. ALAN also had little effect on clock gene abundance in mature oocytes that were dissected from female *Ae. albopictus* that were reared under short day conditions. Our findings indicate that ALAN may inhibit diapause initiation through the circadian clock in two medically-important mosquitoes.

## Introduction

Light pollution caused by artificial light at night (ALAN) is rapidly increasing in urban environments, and is disrupting the light patterns that diverse organisms have used for millennia to regulate their daily and seasonal biology^1–3^. For example, ALAN disrupts the daily timing of singing^4^, advances plant growing season and mean flowering date^5^, and causes orb-web spiders to mature earlier^6^. Further, ALAN inhibits overwintering dormancy, known as diapause, in the horse-chestnut leafminer, *Cameraria ohridella*^7^, the flesh fly, *Sarcophaga similis*^8^, the tiger mosquito, *Aedes albopictus*^9^, and the Northern house mosquito, *Culex pipiens*^10^.

Diapause is a state of developmental arrest that allows insects to survive unfavorable environmental conditions^11,12^. For temperate insects, this largely results in physiological changes that allow insects to survive low temperatures and food scarcity that occur during winter^13,14^. Diapause in temperate insects is triggered primarily by short photoperiods, which are reliable indicators that seasons are changing^15^. Diapause is typically confined to specific life stages within an insect species but there are examples of insects that diapause in the embryonic, pharate first instar, larval or nymphal, pupal, and adult stages^16^. For example, within mosquitoes, different species diapause as eggs (e.g. *Aedes triseriatus*), larvae (e.g., *Wyeomyia smithii*), or as adults (e.g. *Culex restuans*)^17^.

The Northern house mosquito, *Cx. pipiens*, is a major vector of West Nile virus and females enter an adult reproductive diapause in response to short daylengths^18,19^. Diapause in this species is characterized by a lack of host-seeking behavior, arrested egg follicle development, and accumulation of lipid reserves^20,21^ and is induced by a shutdown of juvenile hormone (JH) production^22^. In contrast, maternal exposure to short daylengths induces females of the tiger mosquito, *Ae. albopictus*, to produce eggs that diapause as pharate first instar larvae within the chorion of the egg^23^. Like diapausing females of *Cx. pipiens*, diapausing embryos of *Ae. albopictus* contain more lipid and are also more resistant to desiccation^24,25^. Unlike *Cx. pipiens*, the hormonal regulation of diapause in *Ae. albopictus* is not well understood. However, decreased JH synthesis has also been implicated in diapausing pharate larvae of this species^26^.

Although we do not fully understand how insects translate changes in daylength to downstream hormonal responses, the circadian clock has been implicated in the regulation of diapause in several insect species^27–29^. The insect circadian clock has been extensively characterized in *Drosophila melanogaster* and functions as a set of interlocking negative and positive feedback loops that are influenced by the presence of light, and result in daily changes in the abundance of mRNAs and proteins. Circadian clock genes *Clock* (*Clk*) and *cycle* (*cyc*) encode positive transcription factors that form a protein complex that promotes the expression of other clock genes including *period* (*per*) and *timeless* (*tim*)^30^. During the night, or scotophase, TIM forms a heterodimer with PER and translocates into the nucleus to inhibit CLK:CYC activity. In *D. melanogaster*, CRYPTOCHROME1 (CRY1) degrades TIM in the presence of light, thereby blocking nuclear entry of PER^30,31^. However, most insects outside of the higher diptera, including the monarch butterfly *Danaus plexippus*, the red flour beetle *Tribolium castaneum*, and the mosquitoes *Cx. quinquefasciatus* and *Ae. aegypti*, also possess CRYPTOCHROME2 (CRY2), sometimes referred to as mammalian-type CRYPTOCHROME, that also suppresses CLK:CYC^32–34^. *Par domain protein 1* (*pdp1*) forms another feedback loop that regulates expression of *Clk* in *D. melanogaster* and therefore may regulate expression of *cyc* in other insects where *cyc* transcripts oscillate throughout the day^34–36^. *Pdp1* is also constitutively upregulated in diapausing females of *Cx. pipiens* and knocking down *Pdp1* expression decreases lipid accumulation in diapausing females^35^. The connection between the circadian clock and the diapause response has not been examined in *Ae. albopictus*, although the daily expression profiles of clock genes and proteins have been characterized in *Ae. aegypti*, a closely-related tropical species that does not enter diapause^34,37^. Our previous work has demonstrated that functional circadian clocks are needed to initiate diapause in *Cx. pipiens*^36^. Notably, insect circadian clocks show a high level of homology especially among non-drosophilid insects^38,39^.

ALAN significantly impacts circadian rhythms across many organisms including humans^40^. ALAN also alters the activity patterns of various arthropods by altering locomotor levels and inducing blood-feeding in diurnal *Ae. aegypti*^2,41^. Additionally, our past work has found that ALAN slightly (but not significantly) increases overall activity levels in short-day reared *Cx. pipiens*, and significantly reduces activity levels in long-day reared female mosquitoes^42^. Similarly, another study found that ALAN generally reduced locomotor activity in long-day reared in *Cx. pipiens* f. *molestus*, a subspecies that does not enter diapause^43^. Short-term exposure to ALAN increases *per* and *cry2* transcript abundance in crickets of *Gryllus bimaculatus*^44^ and alters expression of several core circadian clock genes including *per*, *tim*, *cry2*, *cyc*, and *Clk* in *Cx. pipiens* f. *molestus*^43^. However, the effects of ALAN exposure on circadian rhythmicity in diapausing insects has not been studied. Moreover, it is unclear how chronic ALAN (i.e., light pollution that is present for the entire scotophase and throughout development) affects the insect circadian clock. As *Cx. pipiens* and *Ae. albopictus* are generally confined to a single water container as larvae and given that ALAN is largely ubiquitous in urban environments where these species reside^45,46^, measuring how long-term exposure to ALAN affects clock gene abundance is ecologically relevant and could reveal the mechanism by which ALAN is able to disrupt both daily and seasonal changes in mosquito behavior and physiology.

The objective of this study is to determine whether ALAN alters the expression of the circadian clock during diapause in two mosquito species that use different diapause strategies. We hypothesized that ALAN interferes with normal cycling of core circadian clock genes and therefore inhibits diapause entry. We tested this hypothesis by rearing mosquitoes in short-day, diapause-inducing conditions in the presence and absence of ALAN, and then characterizing the daily expression profiles of core circadian clock genes (*cyc*, *Clk, per*, *tim*, *cry2*, *Pdp1* and *cry1*) in the heads of adult females in two mosquito species, *Cx. pipiens* and *Ae. albopictus*, and mature oocytes from the latter.

## Materials and methods

### *Culex pipiens* rearing

A colony of *Cx. pipiens*, established from field-collected egg rafts in Columbus, OH in 2013 (Buckeye strain) were maintained in the laboratory as previously described^42^. First instar larvae (ALAN−) were reared in diapause-inducing conditions (11.5 h light, 12.5 h dark, 20 °C) in growth chambers with approximately 19,000 lx during “daytime” as previously described^10^, and no additional lights during the scotophase (0 lx). A second group of first instar larvae (ALAN+) were reared in a chamber with the same level of light during the photophase but was also fitted with a “warm white” (3000 K) LED (manufactured by Westek Lighting, MRGO-L12W-N1), providing approximately 5 lx of light during “nighttime.” The LED fixture was turned on for the entirety of scotophase and throughout mosquito development. Our previous work determined that this photoperiod and temperature induces diapause at a high incidence in this population when ALAN is absent, whereas exposure to ALAN in these conditions causes female mosquitoes to avert diapause^10^.

Larvae were reared in clear plastic containers (220 larvae per container, 450 mL) to allow for full light penetration and placed within either the ALAN+ or ALAN− incubators. All containers were rotated daily in the growth chamber and larvae were fed ground Tetramin fish flakes. Upon pupation, all pupae were transferred to clear plastic cages (15 × 24 × 20 cm) and allowed to emerge as adults that were provided 10% sucrose solution and kept in their respective ALAN+ and ALAN− incubators.

### Expression profiles of circadian clock genes in *Culex pipiens*

Seven days after peak adult emergence, ALAN+ and ALAN− females of *Cx. pipiens* were collected as at 4 h intervals starting at ZT1 and ending at ZT21, where ZT0 indicates when lights turn on. At each collection timepoint females were euthanized at − 80 °C, then decapitated on dry ice. Total RNA was extracted from mosquito heads (n = 10 heads/biological replicate, 5 replicates per timepoint) using TRIzol reagent (Invitrogen) with slightly modified protocols to accommodate low sample volume^36^. RNA was then further purified using a Lithium Chloride precipitation protocol as previously described^35^. RNA quantity and purity was measured using a Nanodrop spectrophotometer (Nanodrop Products, Wilmington, DE, USA).

The Maxima First Strand cDNA Synthesis Kit for RT-qPCR with DNase (Thermo Fisher Scientific, Waltham, MA, USA) was used to both remove genomic DNA contaminants and synthesize cDNA using 250 ng of total RNA from each biological replicate. cDNA was then diluted fivefold for use in quantitative Real Time PCR (qPCR) reactions. All reactions were performed in triplicate in a 96-well plate using a BioRad CFX Connect Real Time PCR Detection System. The reactions contained 5 μL of Luna Universal qPCR Master Mix (New England Biolabs, Ipswich, MA, USA), 250 nM of each primer, and 1 μL of sample cDNA (total volume = 10 μL). Primer sequences for *cyc*, *Clk*, *per*, *tim*, *cry2*, and reference gene *Rp49* were the same as those used by Gentile et al.^34^ and previously used in *Cx. pipiens*^36^. Primer sequences for *Pdp1*, and reference gene *RpL19* were also previously published^35^.

The cycle threshold (CT) of each biological replicate was averaged across technical replicates, ensuring that the standard deviation of all technical replicates was < 0.2. The resulting CT value for each gene of interest (*cyc*, *Clk*, *per*, *tim*, *cry2*, and *Pdp1)* was normalized to the geometric mean of the CT values for the two reference genes (*Rp49* and *RpL19*) using the 2^−ΔCT^ method^47^. Due to the large number of samples (30 ALAN− and 30 ALAN+ replicates) we were unable to run all samples on a single 96-well plate. Therefore, we ran a subset of ALAN− and ALAN+ samples on both plates to account for plate-to-plate variation. We examined these samples as previously described^35^ and determined that we did not need to apply a plate correction factor as no systematic plate-to-plate variation was detectable.

### *Aedes albopictus* rearing

*Ae. albopictus* were hatched from F1 eggs from a colony that originated from urban Saint Louis, MO USA in 2020. Eggs were submersed for 36 h in a 0.35 g/L Difco Nutrient Broth solution (Becton, Dickenson, and Company). Larvae were sieved and reared in clear plastic group pans with 220 larvae and 2.2 L deionized water each and fed Tetramin fish flakes. Each larval pan was randomly assigned to either the ALAN+ or ALAN− treatment with six pans and a total of 1320 larvae per treatment. Each treatment was housed on a single shelf in the appropriate environmental chamber in the same photoperiod and temperature that were used to rear *Cx. pipiens*. As with *Cx. pipiens*, during the dark phase, mosquitoes in the ALAN− chamber had 0 lx while those in the ALAN+ chamber were exposed to 3–6 lx depending on their position on the shelf. All pans were rotated on the shelf daily. Pupae were plucked from the group pans and held in groups of no more than 5 in 30 mL glass vials plugged with cotton to contain emerged adults. Adults were transferred daily, with a roughly 1:1 sex ratio, into 1.9 L paper food containers with netting tops and given access to a 10% sugar solution. Both pupae and adults were held in the ALAN+ or ALAN− environmental chamber. All adults emerged within a six-day period.

### Validation of diapause incidence in *Aedes albopictus*

As these conditions have not been previously established to initiate diapause in *Ae. albopictus* and to confirm that ALAN does inhibit diapause in these conditions, multiple females of *Ae. albopictus* from the ALAN− and ALAN+ treatment groups were randomly selected to determine diapause incidence. These females were housed in 30.5 × 30.5 × 30.5 cm group cages and given sugar water and a black plastic oviposition cup, lined with seed germination paper and filled with water that was infused with oak leaves to stimulate egg laying. Egg papers were dried and stored in a humidified box in their appropriate ALAN+ or ALAN− chamber for 10 days to complete embryonation. Oviposition cups were checked and any larvae were counted. The eggs were then stimulated to hatch in 0.35 g/L Difco Nutrient Broth solution on two separate occasions to ensure all non-diapausing embryos hatched. All eggs were then bleached and examined under a dissecting microscope to count viable embryos that did not hatch and were therefore assumed to be in diapause^48^.

### Expression profiles of circadian clock genes *Aedes albopictus*

Female *Ae. albopictus* were blood-fed when all adults were at least three-days old. Sugar was removed and adults were offered defibrinated bovine blood twice daily that was tied into a ball in hog intestinal casing, warmed to 60 °C, and placed on the netting on the top of the cages. The mosquitoes were then cold-anesthetized and blood-fed females were sorted and placed in new cages with fresh sugar. Blood feeding continued for three days with blood-fed females housed in groups by the date they fed in the chamber corresponding to their treatment group. Five days after blood-feeding, females were sacrificed at 4 h intervals over a 24 h-period as previously described^35^, where ZT0 indicates when lights turn on. Euthanized females were stored dry in microcentrifuge tubes at − 80 °C. These females were forced to retain their oocytes for later dissection. Female *Ae. albopictus* are reluctant to lay their eggs in small individual cages and will retain a large number, or all of their egg clutch in these conditions (Westby, *personal observation*). The large number of females needed for proper replication prohibited us from using larger cages. Therefore, the decision was made to measure clock gene expression in the mature oocytes rather than fertilized eggs, thus ensuring that all oocytes per replicate were collected from a single female.

Female mosquitoes that had been sacrificed and stored at − 80 °C and not used to determine diapause incidence were partitioned into 5 biological replicates each containing 8 females per collection time point. Heads and oocytes were dissected from frozen mosquitoes on a slide placed on an ice tray. The heads and oocytes were pooled into 1.5 mL microcentrifuge tubes kept on ice. All forceps and slides were thoroughly cleaned with RNase Away between head and oocytes dissections on an individual female and between females. Once all the tissues were harvested, 100 μL of TRIzol was added to each tube and samples were returned to a − 80 °C freezer for storage. Total RNA was isolated from 5 biological replicates of maternal heads or mature oocytes that were collected at each sampling time point (ZT1, 5, 9, 13, 17, and 21) and under each light treatment (n = 30 samples per life stage and light treatment, 120 total samples) using TRIzol reagent as described above. Isolated RNA was then purified and cleaned using the Zymo RNA Clean & Concentrate Kit (Zymo Research, Irvine, CA, USA). RNA purity was assessed using a Nanodrop spectrophotometer (Nanodrop Products, Wilmington, DE, USA). For maternal heads, 3 biological replicates were discarded due to low RNA yield resulting in 4–5 replicates per timepoint. After DNase treatment, 100 ng of RNA from both maternal heads and mature oocytes was used to synthesize cDNA using the Maxima First Strand cDNA Synthesis Kit for RT-qPCR with DNase (Thermo Fisher Scientific, Waltham, MA, USA). The resulting cDNA was diluted fivefold.

We designed primers for core circadian clock genes in *Ae. albopictus* (Supplementary Information; Table S1). For *timeless* (JN559769.1) and *period* (JN559770.1), primers were designed using previously published gene sequences^49^. For all other genes (*cyc*, *Clk*, *cry1*, *cry2*), we found putative *Ae. albopictus* gene sequences by performing BLAST searches on the *Ae. albopictus* genome in GenBank (Aalbo_primary.1) using homologous gene sequences from *Cx. pipiens* or *Ae. aegypti* (Supplementary Information; Table S1). All new primers were evaluated with standard curves to ensure they met efficiency criteria consistent with MIQE guidelines^50^ and melt curves to ensure that a single PCR product was produced (Supplementary Information; Table S2). We used previously published primers for reference genes *RpL34*, *RpL32*, and *RpS17*^25,51^.

The abundance of each clock transcript or reference gene within biological replicate was measured with qPCR as described above, except that the relative abundance of each circadian clock gene within maternal heads was normalized to the geometric mean of the three reference genes (*RpL34*, *RpL32*, and *RpS17*) within the same biological replicate using the 2^−ΔCT^ method^47^. For mature *Ae. albopictus* oocytes, *RpS17* was deemed unacceptable as a reference gene due to differences in CT values between light treatments. Therefore, CT values were normalized to the geometric mean of only *RpL34* and *RpL32*. A subset of ALAN+ and ALAN− samples from maternal heads or oocytes were run on both plates per tissue to ensure that no plate-to-plate variation occurred.

### Statistical analyses

All statistical analyses were performed in R 4.0.4 (R Core Team, 2021). We ensured that the relative abundance of the reference genes did not vary across daily time or light treatment using a two-way ANOVA for ZT and light treatment within each species and life stage (Supplementary Information; Table S3).

For each species and tissue, we tested the difference in relative abundance of circadian clock genes throughout the day using separate linear models for ALAN+ and ALAN− data with collection timepoint (ZT) as a factor. We evaluated the normality of the model by examining plots of the residuals and using a Shapiro–Wilks test on the residuals and tested for homogeneity of variance using a Score Test for Non-Constant Error Variance (*car* package)^52^. When the linear model did not satisfy normality or variance tests, a logarithmic transformation was applied to the data or a Generalized Linear Model was used (Supplementary Information; Table S4). We evaluated each model for potential outliers and influential points using a Bonferroni Outlier Test (*car* package)^52^ and removed them when indicated. We performed Type II Analysis-of-Variance test to generate F test statistics (car package)^52^ and to determine whether relative mRNA abundance varied through the day. Significant differences in mRNA abundances across timepoints were generated using estimated marginal means (*emmeans* package)^53^. We identified genes that cycled throughout the day as those that had a significant difference in the contrasts, representing mRNA abundance, between ZT5 (approximately middle of photophase, 5 h after lights on) and ZT17 (approximately middle of scotophase, 5.5 h after lights off)^54^. Differences in the mRNA abundance between all time points for each gene, species, tissue, and light treatment are reported in Table S5, S6, and S7 (Supplementary Materials).

To test whether ALAN altered gene expression, we used linear models where ZT, light treatment, and their interaction were all used as factors (referred to as full models). We evaluated the normality and equal variance of each full model as described above. When the model did not meet normality and variance assumptions, a logarithmic transformation was applied or a Generalized Linear Model was used (Supplementary Information, Table S4). As above, we performed a Type II Analysis-of-Variance test to generate F-test statistics for each variable in the model (*car* package)^52^. We used simple contrasts to obtain pairwise comparisons of relative mRNA abundance between ALAN+ and ALAN− treatments at each timepoint using estimated marginal means (*emmeans* package)^53^.

## Results

### Validation of diapause incidence and ALAN-induced aversion in *Ae. albopictus*

Diapause incidence was calculated as the proportion of eggs in diapause (e.g., the number of unhatched embryos/the total viable embryos [hatched and unhatched]). Diapause incidence for the ALAN− treatment was 99.6% (1257 unhatched embryos/1262 total embryos) and was 21.8% for the ALAN+ treatment (231 unhatched embryos/1056 total embryos).

### Expression profiles of core circadian clock genes

Several core circadian clock genes varied throughout the day in adult females of *Cx. pipiens*, maternal females of *Ae. albopictus*, and mature oocyte tissue from *Ae. albopictus* under both ALAN− and ALAN+ conditions. ALAN exposure also significantly altered the transcript abundance and/or daily expression profile of several core circadian clock genes in both mosquito species and in different life stages of *Ae. albopictus*, both overall and in a time-dependent manner. Below we describe the daily expression profile of each core circadian clock gene in the heads of female *Cx. pipiens*, maternal *Ae. albopictus*, and mature oocytes of *Ae. albopictus*, as well as how ALAN exposure affected the overall abundance of each core clock gene.

### Relative abundance of *cycle* transcripts

**Figure 1:**
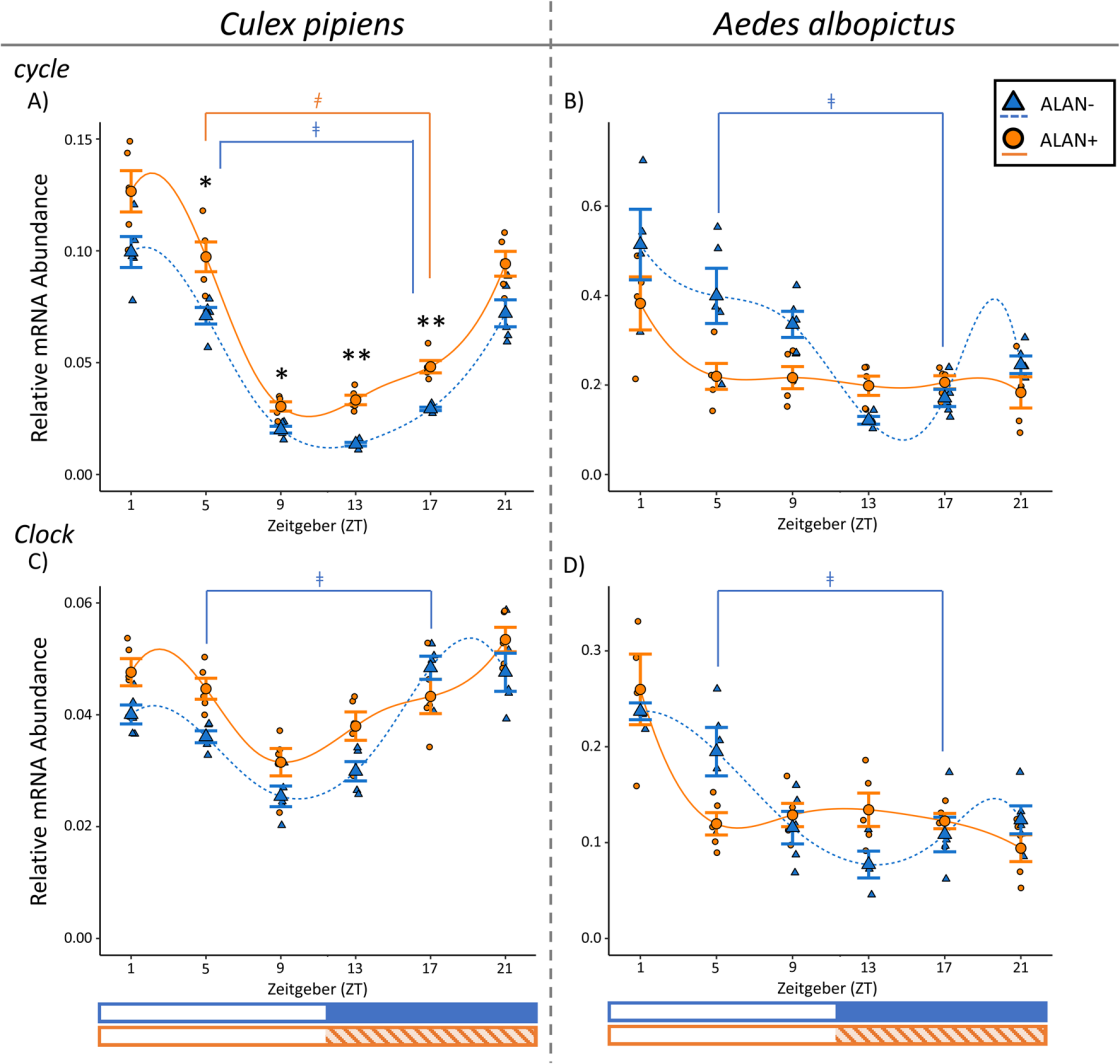
Daily mRNA expression profiles of core circadian clock genes *cycle* (**A**, **B**) and *Clock* (**C**, **D**) in the heads of adult females of *Cx. pipiens* (**A**, **C**), and the heads of adult females of *Ae. albopictus* (**B**, **D**). Gene expression in short-day reared (11.5:12.5 L:D, 20 °C), diapausing control mosquitoes (ALAN−) are shown in blue triangles and dashed lines, while gene expression in those exposed to ALAN under the same conditions (ALAN+) are shown in orange circles and solid lines. Smaller markers represent relative mRNA abundance in biological replicates consisting of heads from 8 to 10 females; and the large marker and bars represent mean ± standard error of 4–5 biological replicates. Spline curves were fit to the data using R. Photophase (white bar) and scotophase (colored bar) are represented in Zeitgeber (ZT) under the x-axis, with exposure to ALAN shown in the shaded orange bar. Significant differences between ALAN-exposed mosquitoes and diapause control at each sampled Zeitgeber (ZT) are indicated with an asterisk (*p < 0.05, **p < 0.0001), and significant differences between ZT5 and ZT17 (daily cycling of mRNA expression) within each rearing condition are represented with a ǂ (*p* < 0.05; Two-Way Linear Model with simple contrasts using Estimated Marginal Means).

The relative mRNA abundance of *cyc* in *Cx. pipiens* significantly changed throughout the day in both ALAN− (F = 154.37, *p* < 0.0001; ZT5 vs ZT17: t ratio = 9.59, *p* < 0.001) and ALAN+ (F = 85.65, *p* < 0.001; ZT5 vs ZT17: t ratio = 7.55, *p* < 0.001; Fig. 1A), with peak abundance occurring shortly after the onset of photophase. However, in ALAN− *Cx. pipiens*, lowest mRNA abundance occurred shortly after the onset of scotophase (ZT13) and in ALAN+ mosquitoes lowest mRNA abundance occurred shortly before scotophase (ZT9). The relative expression of *cyc* was elevated in ALAN+ mosquitoes during photophase (ZT5: t ratio = 3.39, *p* = 0.035; ZT9: t ratio = 4.55, *p* = 0.001) and scotophase (ZT13: t ratio = 9.76, *p* < 0.001; ZT17: t ratio = 5.33, *p* = 0.0001; Fig. 1A). Therefore, ALAN exposure significantly increased *cyc* mRNA abundance in *Cx. pipiens* both on average as a main effect (F = 136.35, *p* < 0.001) and in a time-dependent manner (F = 7.00, *p* < 0.0001).

Relative *cyc* mRNA abundance significantly changed throughout the day for maternal *Ae. albopictus* under both ALAN− (F = 17.38, *p* < 0.001) and ALAN+ conditions (F = 3.05, *p* = 0.029). In ALAN− mosquitoes peak abundance occurred after the onset of photophase in ALAN− mosquitoes, and these mosquitoes showed evidence of rhythmic *cyc* expression (ZT5 vs ZT17: t ratio = 4.89, *p* = 0.0009). However, ALAN+ individuals did not exhibit oscillation of *cyc* expression (Fig. 1B; ZT5 vs ZT17: t ratio = 0.20, *p* = 1.00), and all differences in *cyc* mRNA abundance over time in ALAN+ maternal *Ae. albopictus* were driven by elevated relative mRNA abundance at ZT1 (ZT1 vs ZT21: t ratio = 3.68, *p* = 0.014). The full model revealed that ALAN exposure significantly affected *cyc* expression in maternal *Ae. albopictus* as a main effect (F = 5.32, *p* = 0.026) and as an interaction with time (F = 4.78, *p* = 0.001), although there were no significant differences in the relative abundance of *cyc* mRNA transcripts between ALAN+ and ALAN− samples at specific timepoints (Fig. 1B).

Relative *cyc* mRNA abundance did not vary throughout the day in *Ae. albopictus* oocytes in either the ALAN− (F = 1.59, *p* = 0.20) or ALAN+ (F = 1.67, *p* = 0.18; Supplemental Materials, Fig. S1). Additionally, ALAN exposure did not alter *cyc* abundance in *Ae. albopictus* oocytes as a main effect (F = 0.22, *p* = 0.64) nor as an interaction with time (F = 1.16, *p* = 0.34; Supplemental Materials, Fig. S1).

### Relative abundance of *clock* transcripts

In *Cx. pipiens*, relative *Clk* mRNA abundance significantly changed throughout the day in ALAN− mosquitoes (F = 19.73, *p* < 0.001) and ALAN+ mosquitoes (F = 9.64, *p* < 0.001; Fig. 1C). Notably, *Clk* mRNA abundance was significantly lower during the middle of photophase relative to the middle of scotophase and therefore met our criteria for rhythmic expression in ALAN− mosquitoes (ZT5 vs ZT17: t ratio = − 4.17, *p* = 0.004) but not ALAN + mosquitoes (ZT5 vs ZT17: t ratio = 0.40, *p* = 1.00; Fig. 1D). For both light conditions, lowest *Clk* mRNA abundance occurred at the end of photophase (ZT9). ALAN significantly increased *Clk* expression in *Cx. pipiens* on its own (Fig. 1C; F = 15.45, *p* = 0.0003) and in a time-dependent manner (F = 2.56, *p* = 0.039) although there was no significant difference in *Clk* mRNA abundance between ALAN+ and ALAN− mosquitoes at any specific timepoint.

Similarly, *Clk* relative mRNA abundance changed throughout the day in adult females of *Ae. albopictus* that were exposed to ALAN− (F = 10.48, *p* < 0.0001) and ALAN + conditions (F = 6.94, *p* = 0.0004; Fig. 1D). We detected daily oscillation of *Clk* mRNA in ALAN− mosquitoes (ZT5 vs ZT17: t ratio = 3.55, *p* = 0.02), but not in ALAN+ mosquitoes (ZT5 vs ZT17: t ratio = − 0.20, *p* = 1.00). ALAN exposure did affect the overall abundance of *Clk* mRNA in maternal *Ae. albopictus* as an interaction with time (Fig. 1D; F = 3.79, *p* = 0.006), but post-hoc simple contrasts did not show any significant differences at any specific timepoint.

In the oocytes from *Ae. albopictus* we found that *Clk* mRNA transcript abundance varied through the day (but did not oscillate) in ALAN− *Ae. albopictus* oocytes (F = 4.62, *p* = 0.001; ZT5 vs ZT17: t ratio = 2.55, *p* = 0.13), where peak *Clk* mRNA abundance occurred at ZT9 and minimum abundance occurred at ZT17 (Supplemental Materials, Fig. S1; ZT9 vs ZT13: t ratio = 3.08, *p* = 0.037; ZT9 vs ZT17: t ratio = 3.27, *p* = 0.023). The abundance of *Clk* mRNA transcripts did change throughout the day in ALAN+ oocytes (F = 2.61, *p* = 0.05), but did not oscillate (ZT5 vs ZT17: t ratio = 1.19, *p* = 0.84). ALAN did not affect the abundance of *Clk* mRNA transcripts in *Ae. albopictus* oocytes on its own (F = 0.27, *p* = 0.60) nor in a time-dependent manner (F = 0.75, *p* = 0.59).

### Relative abundance of *period* transcripts

**Figure 2:**
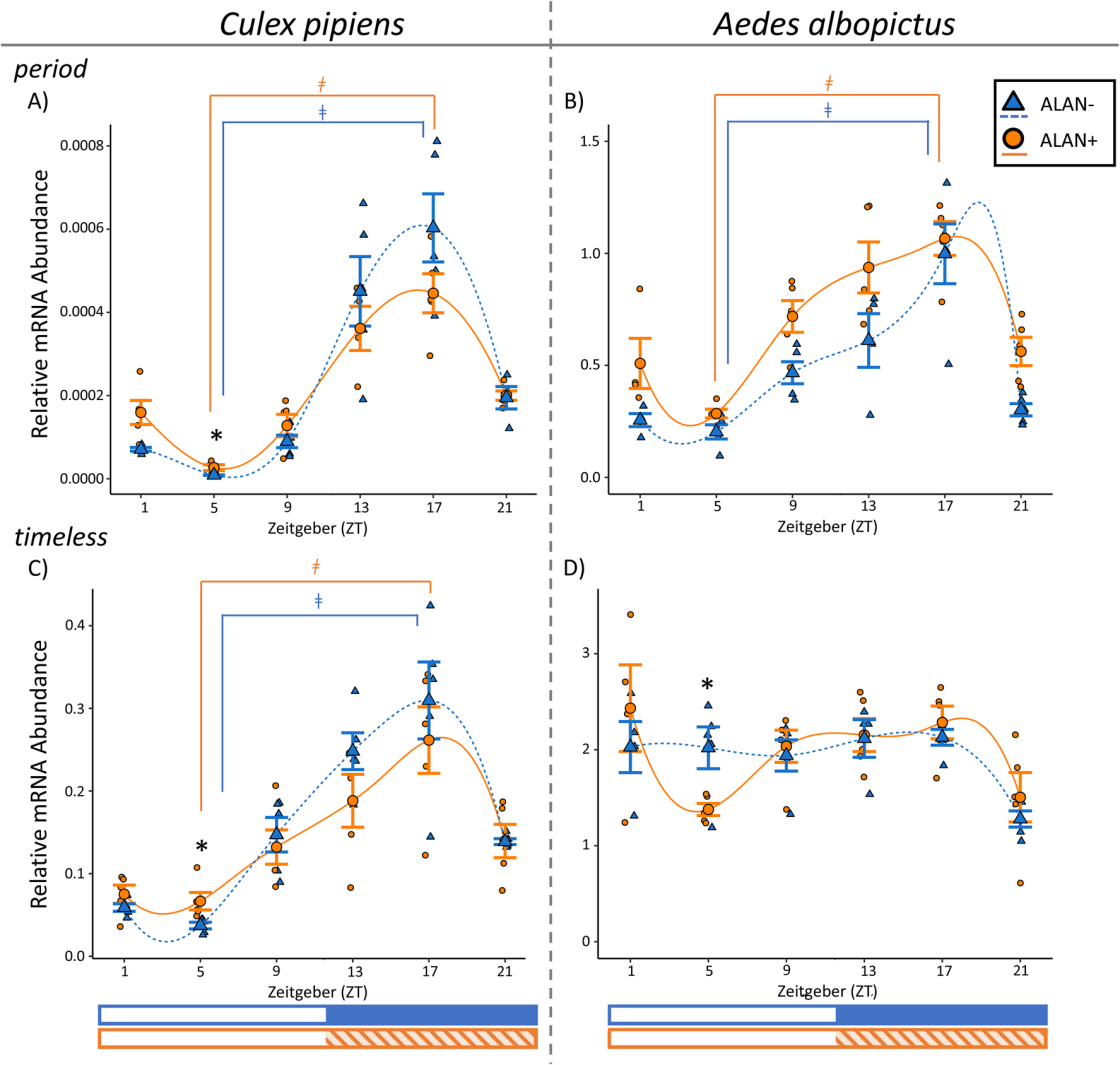
Daily mRNA expression profiles of core circadian clock genes *period* (**A**, **B**) and *timeless* (**C**, **D**) in the heads of adult females of *Cx. pipiens* (**A**, **C**), and the heads of adult females of *Ae. albopictus* (**B**, **D**). Gene expression in short-day reared (11.5:12.5 L:D, 20 °C), diapausing control mosquitoes (ALAN−) are shown in blue triangles and dashed lines, while gene expression in those exposed to ALAN under the same conditions (ALAN+) are shown in orange circles and solid lines. Smaller markers represent relative mRNA abundance in biological replicates consisting of heads from 8 to 10 females; and the large marker and bars represent mean ± standard error of 4–5 biological replicates. Spline curves were fit to the data using R. Photophase (white bar) and scotophase (colored bar) are represented in Zeitgeber (ZT) under the x-axis, with exposure to ALAN shown in the shaded orange bar. Significant differences between ALAN-exposed mosquitoes and diapause control at each sampled Zeitgeber (ZT) are indicated with an asterisk (*), and significant differences between ZT5 and ZT17 (daily cycling of mRNA expression) within each rearing condition are represented with a ǂ (*p* < 0.05; Two-Way Linear Model with simple contrasts using Estimated Marginal Means).

Relative *per* expression varied throughout the day in ALAN− *Cx. pipiens* (Fig. 2A; F = 93.27, *p* < 0.0001), and showed robust cycling (ZT5 vs ZT17: t ratio = − 19.04, *p* < 0.0001). Under ALAN exposure, *per* expression still varied rhythmically throughout the day in *Cx. pipiens* (Fig. 2A; F = 24.28, *p* < 0.0001; ZT5 vs ZT17: z ratio = − 11.43, *p* < 0.0001). Relative mRNA abundance of *per* was significantly altered by ALAN exposure (Light Treatment: F = 5.88, *p* = 0.019; Light Treatment × Time: F = 3.48, *p* = 0.010), and *per* mRNA abundance was significantly higher in ALAN+ mosquitoes at ZT5 (Fig. 2A; t ratio = 3.41, *p* = 0.034; twofold increase).

ALAN had a similar effect on the expression profile of *per* in the heads of female *Ae. albopictus*. In maternal *Ae. albopictus* that were not exposed to ALAN, *per* transcripts also showed significant changes and evidence of cycling (Fig. 2B; F = 14.46, *p* < 0.0001; ZT5 vs ZT17: t ratio = − 7.47, *p* < 0.0001), with peak *per* mRNA abundance occurring in the middle of scotophase. In the presence of ALAN, *per* expression continued to cycle (Fig. 2B; F = 13.28, *p* < 0.0001; ZT5 vs ZT17: t ratio = − 7.03, *p* < 0.0001). ALAN significantly increased *per* expression in maternal *Ae. albopictus*, but only as a main effect of the model (F = 30.51, *p* < 0.0001) and there were not any specific sampling timepoints when the abundance of *per* mRNA significantly differed between ALAN+ and ALAN− mosquitoes.

We observed a different trend in *per* expression in *Ae. albopictus* oocytes, where in the absence of ALAN *per* significantly changed throughout the day (Supplemental Materials, Fig. S1; F = 5.15, *p* = 0.003), but peak mRNA abundance occurred at ZT9 and we found no evidence of cycling (ZT5 vs ZT17: t ratio = − 0.199, *p* = 1.00). Notably, *per* did not show any daily changes in mRNA abundance in *Ae. albopictus* oocytes that were exposed to ALAN (F = 0.71, *p* = 62). However, ALAN had no effect on *per* expression in *Ae. albopictus* oocytes (Light Treatment: F = 0.63, *p* = 0.43; Light Treatment × Time: F = 0.33, *p* = 0.90).

### Relative abundance of *timeless* transcripts

Relative *tim* expression oscillated throughout the day in the heads of *Cx. pipiens* that were reared in both ALAN− (F = 45.45, *p* < 0.0001; ZT5 vs ZT17: t ratio = − 12.25, *p* < 0.0001) and ALAN+ conditions (F = 9.02, *p* < 0.0001; ZT5 vs ZT17: t ratio = − 5.53, *p* = 0.0001) *Cx. pipiens* with peak expression occurring in the middle of scotophase (Fig. 2C). However, relative *tim* mRNA abundance was 80% higher at ZT5 in ALAN+ relative to ALAN− *Cx. pipiens* (t ratio = 3.97, *p* = 0.002), and ALAN altered *tim* expression in *Cx. pipiens* as a main effect (F = 19.53, *p* < 0.001).

Relative *tim* expression varied throughout the day in both ALAN− (F = 3.95, p = 0.010) and ALAN+ (F = 3.59, *p* = 0.015) maternal *Ae. albopictus*, but we did not detect daily oscillation in either ALAN− (ZT5 vs ZT17: t ratio = − 0.58, *p* = 0.99) nor ALAN+ conditions (ZT5 vs ZT17: t ratio = − 2.91, *p* = 0.07; Fig. 2D). Relative expression of *tim* did not vary throughout the day in *Ae. albopictus* oocytes in ALAN− (F = 1.35, *p* = 0.28) nor ALAN+ conditions (F = 1.39, *p* = 0.26; Supplemental Materials, Fig. S1). Additionally, ALAN had no effect on the relative mRNA abundance of *tim* in mature oocytes from *Ae. albopictus* (Light Treatment: F = 0.53, *p* = 0.47; Light Treatment × Time: F = 0.44, *p* = 0.82) but significantly decreased *tim* mRNA abundance in maternal *Ae. albopictus* at ZT5 (Light Treatment: F = 0.94, *p* = 0.34; Light Treatment x Time: F = 4.61, *p* = 0.002; z ratio = − 3.36, *p* = 0.022).

### Relative abundance of *cryptochrome 2* transcripts

**Figure 3:**
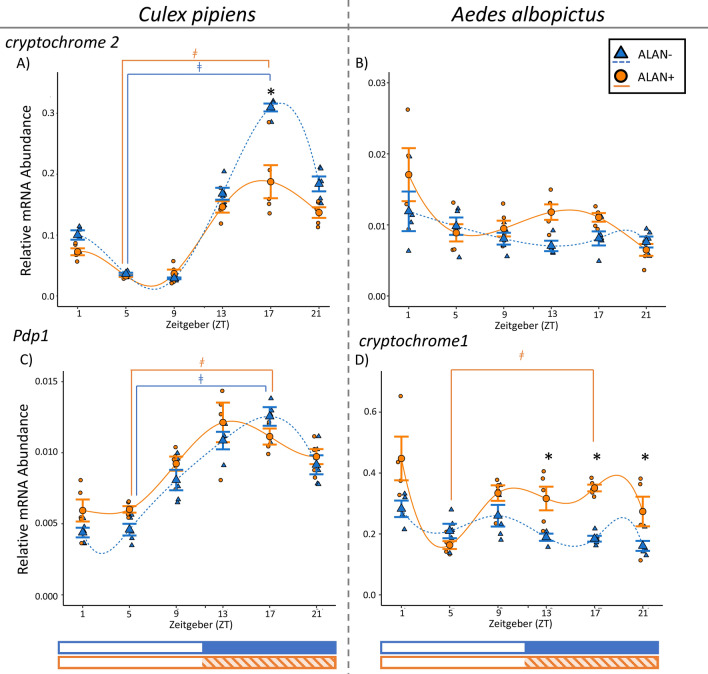
Daily mRNA expression profiles of core circadian clock genes *Par domain protein 1* (*Pdp1*; **C**) *cryptochrome 1* (**D**) and *cryptochrome 2* (**A**, **B**) in the heads of adult females of *Cx. pipiens* (**A**, **C**), and the heads of adult females of *Ae. albopictus* (**B**, **D**). Gene expression in short-day reared (11.5:12.5 L:D, 20 °C), diapausing control mosquitoes (ALAN−) are shown in blue triangles and dashed lines, while gene expression in those exposed to ALAN under the same conditions (ALAN+) are shown in orange circles and solid lines. Smaller markers represent relative mRNA abundance in biological replicates consisting of heads from 8 to 10 females; and the large marker and bars represent mean ± standard error of 4–5 biological replicates. Spline curves were fit to the data using R. Photophase (white bar) and scotophase (colored bar) are represented in Zeitgeber (ZT) under the x-axis, with exposure to ALAN shown in the shaded orange bar. Significant differences between ALAN-exposed mosquitoes and diapause control at each sampled Zeitgeber (ZT) are indicated with an asterisk (*), and significant differences between ZT5 and ZT17 (daily cycling of mRNA expression) within each rearing condition are represented with a ǂ (*p* < 0.05; Two-Way Linear Model with simple contrasts using Estimated Marginal Means).

The relative abundance of *cry2* mRNA cycled in *Cx. pipiens*, both in the absence and presence of ALAN (ALAN−: F = 253.84, *p* < 0.0001, ZT5 vs ZT17: t ratio = − 25.60, *p* < 0.0001; ALAN+: F = 47.28, *p* < 0.0001, ZT5 vs ZT17: t ratio = − 11.02, *p* < 0.0001; Fig. 3A), with peak expression occurring in the middle of scotophase. Exposure to ALAN also significantly decreased relative *cry2* expression in *Cx. pipiens* in a time-dependent manner (Light Treatment: F = 15.73, *p* = 0.0002; Light Treatment × Time: F = 3.73, *p* = 0.006). Specifically, relative *cry2* expression was 0.4-fold lower at ZT17 in ALAN+ mosquitoes relative to ALAN− mosquitoes (t ratio = − 4.31, *p* = 0.002; Fig. 3A).

In contrast, *cry2* mRNA abundance did not change throughout the day in ALAN− maternal *Ae. albopictus* (F = 2.00, *p* = 0.12) but did in ALAN+ females (F = 5.45, *p* < 0.002; Fig. 3B), although it did not oscillate as there were no significant differences in the abundance of *cry2* between the middle of photophase and the middle of scotophase (ZT5 vs ZT17: t ratio = − 1.36, *p* = 0.75). In the ALAN-exposed females, the abundance of *cry2* mRNAs at ZT21 was significantly lower than at ZT1 (t ratio = 4.83, *p* = 0.0009) and ZT13 (t ratio = 3.41, *p* = 0.026). ALAN exposure significantly increased mean *cry2* mRNA abundance in maternal *Ae. albopictus* heads (F = 4.80, *p* = 0.034), but there was no time-dependent effect (F = 2.04, *p* = 0.091).

The mRNA abundance of *cry2* did not change throughout the day in *Ae. albopictus* oocytes that were exposed to either ALAN− (F = 1.80, *p* = 0.15) or ALAN+ conditions (F = 1.61, *p* = 0.20; Supplemental Materials, Fig.S1). Additionally, ALAN did not affect *cry2* expression in *Ae. albopictus* eggs (Light Treatment: F = 2.54, *p* = 0.11; Light Treatment × Time: F = 2.16, *p* = 0.07).

### Relative abundance of *par domain protein-1* transcripts

Relative expression of *Pdp1* oscillated throughout the day in the heads of females of *Cx. pipiens* that were exposed to ALAN (F = 14.04, *p* < 0.0001; ZT5 vs ZT17: t ratio = − 9.33, *p* < 0.0001) and those that were not (F = 14.04, *p* < 0.0001; ZT5 vs ZT17: t ratio = − 5.38, *p* < 0.0001; Fig. 3C), with peak expression occurring at ZT17 for ALAN− mosquitoes and ZT13 ALAN+ mosquitoes. ALAN exposure significantly increased *Pdp1* transcript abundance in *Cx. pipiens* (F = 6.72, *p* = 0.013), but not as an interaction with time (F = 1.39, *p* = 0.25; Fig. 3C).

### Relative abundance of *cryptochrome 1* transcripts

As previous studies demonstrate that *cry1* does not oscillate in *Culex* spp.^34,36^, we only measured the relative abundance of *cry1* transcripts in *Ae. albopictus*. Relative *cry1* mRNA abundance varied throughout the day in maternal *Ae. albopictus* in both light conditions (ALAN−: F = 4.23, *p* = 0.008; ALAN+: F = 6.73, *p* = 0.0005), but only fit our definition for oscillation under ALAN+ (ZT5 vs ZT17: t ratio = − 4.30, *p* = 0.003; Fig. 3D). ALAN also increased expression of *cry1* in maternal *Ae. albopictus* heads (Fig. 3D; Light Treatment: F = 39.84, *p* < 0.0001; Light Treatment × Time: F = 5.65, *p* = 0.0004), such that *cry1* expression was upregulated during scotophase in ALAN+ female heads (ZT13: t ratio = 3.32, *p* = 0.044; ZT17: t ratio = 4.67, *p* = 0.0009; ZT21: t ratio = 4.53, *p* = 0.001). In contrast, *cry1* mRNA abundance did not vary throughout the day in mature oocytes from either ALAN exposed or unexposed *Ae. albopictus* (ALAN−: F = 1.71, *p* = 0.17; ALAN+: F = 1.65, *p* = 0.18 ; Supplemental Materials, Fig. S1). ALAN had no effect on *cry1* expression in *Ae. albopictus* oocytes (Main Effect: F = 1.55, *p* = 022; Interaction with Time: F = 1.37, *p* = 0.25).

## Discussion

We found that exposure to ALAN significantly impacted both the daily expression profiles and the overall abundance of several core circadian transcripts in the heads of adult females of *Cx. pipiens* and *Ae. albopictus*. As these mosquitoes were reared in short-day, diapause-inducing conditions, our results imply that exposure to ALAN broadly alters the circadian clock in two medically-important mosquito species. Our results corroborate past work done in *Cx. pipiens* f. *molestus*, which also found that ALAN significantly altered expression of *cycle*, *Clock*, *period*, *timeless*, and *cryptochrome2*^43^. In contrast to this previous study, we examined how chronic exposure to ALAN throughout development affected the daily expression profile of clock genes.

Interestingly, ALAN did not eliminate rhythmicity of the central circadian clock for most of the genes we measured. Similar results have been reported in tree sparrows and zebra finches^55,56^. As the circadian clock is highly conserved in function across animal taxa^57^, our results indicate that the central circadian clock can continue to function when organisms are exposed to ALAN, but implies that small differences in the abundance of mRNAs in the presence of light may still affect behavioral and physiological components of the diapause program and possibly other seasonal responses in animals. For example, ALAN induces blood-feeding behavior and higher flight activity in short-day reared *Cx. pipiens*, but does not impact the circadian rhythmicity of their behavioral profiles^10,42^. As *Cx. pipiens* are crepuscular and *Ae. albopictus* are diurnal^58–60^, changes in clock gene expression may cause different behavioral modifications in each species. Our observation that *cyc* mRNA transcripts did not oscillate in heads of *Ae. albopictus* in the presence of ALAN suggests that these females may have lost rhythmic changes in locomotor activity, although this will need further investigation. ALAN exposure increased nighttime biting activity in diurnal *Ae. aegypti*^41^, and so similar patterns may occur in *Ae. albopictus*.

Our results illuminate how ALAN may impact the circadian clock. Given that in *D. melanogaster* and other insects, the photo-sensitive form of CRY (CRY1) degrades TIM in the presence of light and thus prevents PER from entering the nucleus^31,32^, it is possible that the presence of ALAN allows for TIM to be degraded throughout the night. If this occurs, PER would not inhibit the activity of CYC and CLK during scotophase. Additionally, in most insects aside from *D. melanogaster*, including monarch butterflies and *Cx. pipens*, a photo-insensitive form of CRY (CRY2) also suppresses CYC:CLK activity independent of PER^32,33^. Since *cry2* was downregulated in the middle of scotophase in *Cx. pipiens* heads in response to ALAN, this could represent another mechanism by which CYC:CLK activity in ALAN-exposed mosquitoes is not being sufficiently suppressed at night.

We found that exposure to ALAN broadly upregulated *cyc* mRNA abundance in *Cx. pipiens*, particularly during the night. Additionally, both *tim* and *per* transcripts were generally lower in the heads of female *Cx. pipiens*. These results both provide evidence toward our mechanistic hypothesis and corroborate past studies in *Cx. pipiens* f. *molestus* that found *cyc* to be upregulated and *per* to be downregulated in the presence of ALAN^43^. Additionally, knockout or knockdown of *per* and *tim* also decreased diapause incidence in the silkworm *Bombyx mori*^61^, and *Cx. pipiens*^36^, indicating that lower levels of these genes are associated with averting diapause. Additionally, we found differences in mRNA abundance of both *cry1* and *cry2*. We found that *cry2* expression was significantly suppressed in *Cx. pipiens* during the middle of scotophase. This finding provides further evidence toward continued CYC:CLK activity during scotophase under ALAN exposure and supports past work that found that knocking down *cry2* causes females of *Cx. pipiens* to avert diapause^36^. Although we did not examine how ALAN exposure affected *cry1* transcript abundance in *Cx. pipiens*, we found that *cry1* mRNA was upregulated specifically during scotophase in female *Ae. albopictus*. As CRY1 is the photo-sensitive form of CRY used to entrain the endogenous circadian clock, this could represent a direct response to the additional light at night^31^. However, our results should be interpreted with some caution as the differences we observed in mRNA abundances may not translate to differences in protein abundances or activity. Future experiments that measure the abundance and activity circadian clock proteins will be necessary in order to determine if ALAN is impacting the circadian clock in this manner.

As previously mentioned, we observed that *cyc* transcripts were constitutively upregulated in short-day reared *Cx. pipiens* that were exposed to low levels of ALAN. Our earlier work also demonstrated that exposure to ALAN induced short-day reared females of *Cx. pipiens* to develop ovaries, imbibe a blood meal, and produce viable offspring^10^. Although the mechanism by which this might occur is unclear, it possible that the CYC transcription factor may directly regulate genes responsible for reproductive development. Specifically, E-box promotor sites to which CYC binds are present within the promotor region of *allatotropin* (CPIJ007896) in *Cx. pipiens*. This is notable because the ALLATOTROPIN neuropeptide stimulates the *corpora allata* to produce JH and thereby stimulate reproductive development in *Cx. pipiens*^62^. Additionally, CYC binds to and regulates *allatotropin*^63^. Therefore, the upregulation of *cycle* transcripts in ALAN-exposed mosquitoes that we observed likely leads to increased CYC protein abundance and/or activity, higher levels of ALLATOPTROPIN and JH to stimulate reproductive development. Future research should, however, investigate whether this the mechanism by which ALAN stimulates reproductive development in short-day reared females of *Cx. pipiens*.

Additionally, we found that clock genes did not oscillate in the mature oocytes of *Ae. albopictus* that were dissected from short-day reared females and that ALAN did not impact the expression of clock genes in this tissue. Like other animals, insects have peripheral clocks in their reproductive tissue that has been largely associated with circadian rhythms in mating, sperm release, and egg-laying^64^. However, despite the lack of oscillation in clock genes in the oocytes we were able to detect mRNAs of all examined clock genes. This consistent with evidence from mammals demonstrating that several circadian clock genes are expressed in unfertilized oocytes, as well as an earlier study in *D. melanogaster* that demonstrates that *per* and *tim* are constitutively expressed in the ovaries where they play an important role in oogenesis^65,66^. Additionally, it is possible that clock genes do not display rhythmic changes in mRNA abundance until after embryogenesis. Eggs of pea aphids that are exposed to Light:Dark cycles exhibit temporal variation in *cyc* expression, but these differences are heavily dampened after being exposed to constant darkness^67^, suggesting that circadian clock gene expression in insect eggs may not be as robust as in larval or adult insects. Future studies on the expression profile of circadian clock genes in eggs of *Ae. albopictus* will determine whether circadian transcripts oscillate in diapausing (or nondiapausing) eggs and whether ALAN disrupts those cycles. Lastly, although we did not find an effect of ALAN on clock gene expression in mature oocytes, other processes may be disrupted. For example, mature oocytes from short-day reared *Ae. albopictus* upregulate *fatty acyl coA elongase* indicating that there may be some differences in gene expression in these tissues prior to fertilization and the development of the diapausing embryo^25^ and these could be disrupted by exposure to ALAN.

Surprisingly, we failed to detect daily oscillations in the abundance of *tim* and *cry2* mRNAs in the heads of adult female *Ae. albopictus*. These results were counter to our expectations as both *tim* and *cry2* are rhythmically expressed in multiple mosquito species including *Cx. quinquefasciatus*, diapausing females of *Cx. pipiens*, as well as in closely-related *Ae. aegypti*^34,36^. Notably, we measured clock gene expression in recently blood-fed females that were ready to oviposit. As *Ae. albopictus* show a clear diel pattern of oviposition^68^, we would predict that these females would display strong rhythmic changes in circadian transcript abundance. Our findings could indicate that loss of rhythmic changes in *tim* and *cry2* mRNA abundance is associated with producing diapausing eggs in *Ae. albopictus*. However, we caution against overinterpretation of these results; it is possible that the oscillation of these clock genes in short-day reared females of *Ae. albopictus* is relatively weak and that high levels of variability in relative mRNA abundance among our samples prevented us from detecting it, or oscillation of mRNAs are desychronized between clock cells in the brain under this condition. Additionally, it is unknown whether *tim* and *cry2* oscillate in *Ae. albopictus* in long-day conditions. *cry2* and/or *tim* show rhythmic expression in four other mosquito species in long-day conditions: *Ae. aegypti*, *Cx. quinquefasciatus*, *Cx. pipiens*, and *Anopheles gambiae*^34,36,69^. However, further experiments that characterize clock gene expression profiles in long-day reared females of *Ae. albopictus* will be necessary to evaluate this.

Another surprising finding that we uncovered is that *Clk* expression oscillated in short day-reared females of *Cx. pipiens* and *Ae. albopictus* in the absence of ALAN. Previous studies demonstrate that *Clk* does not oscillate in *Cx. quinquefasciatus* and *Ae. aegypti*^34^*,* as well as in diapausing females of *Cx. pipiens*^36^, which is inconsistent with our current results. However, Gentile et al.^34^ found that *Clk* transcripts did show temporal variation in abundance in *Cx. quinquefasciatus*, even if they did not oscillate. Notably, the changes in *Clk* abundance between the middle of the photophase and middle of the scotophase in ALAN− *Cx. pipiens* are relatively small (34%), and therefore it is possible that this is a result that is statistically significant but may not be highly biologically relevant. However, *Clk* transcripts have been found to oscillate in the heads of *An. gambiae*^69^. Therefore, it is possible that *Clk* mRNA transcripts may oscillate in additional insects or under specific conditions. Future studies should investigate whether *Clk* mRNA transcripts oscillate, as well as determine how the loss of rhythmic expression in *Clk* abundance in *Cx. pipiens* and *Ae. albopictus* that were exposed to ALAN impacts their daily and seasonal phenotypes.

Although ALAN generally impacted clock gene expression in both *Cx. pipiens* and *Ae. albopictus*, the differences in how ALAN altered the abundance and/or expression profile of specific genes highlights the need to examine responses across species and among insects that differ in their diapause strategies. For example, exposure to ALAN caused broad upregulation of *cyc* mRNA abundance in *Cx. pipiens* with particularly strong differences during early to mid-scotophase. However, ALAN exposure completely eliminated daily *cyc* oscillation in *Ae. albopictus*. The circadian clock has been now linked to the diapause response in insects that enter embryonic, larval or nymphal, and adult diapauses^29,61^. Further, studies have found that ALAN inhibits diapause in insect that diapause as eggs, pupae, and adults^7,9,10^. Our experiments have uncovered similarities and differences in how ALAN affects the circadian clock during diapause in mosquitoes that overwinter as embryos and adults, indicating that ALAN’s ability to inhibit diapause might be due to dysregulation of the core circadian clock. Future studies should examine how ALAN influences the clock in insects that enter larval and pupal diapauses. Further, the circadian clock in insects is not just responsible for seasonal timekeeping and inducing diapause, but regulates behavioral patterns such as hatching, feeding, eclosion, and mating, as well as a wide range of physiological processes including memory, metabolism, and immunity^70^. Therefore, ALAN’s influence on the circadian clock can have many potential impacts that could interfere with an organism’s ability to survive and thrive in the urban environment. Taken together, our results demonstrate that ALAN likely impacts the ability of urban mosquitoes to appropriately measure seasonal time and has important implications for disease transmission and control efforts.

### Supplementary Information

Supplementary Information.

## Data Availability

Data are available at https://figshare.com/s/806b216f2f3ac237382a. Raw qPCR files from each machine run are available upon request to the corresponding author.
