# Supplementary material for: Light pollution disrupts circadian clock gene expression in two mosquito vectors during their overwintering dormancy
Source: Sci Rep. 2024 Jan 29;14:2398. doi: 10.1038/s41598-024-52794-x (PMC10824765; doi:10.1038/s41598-024-52794-x)
Supplement: Supplementary file 1 — Supplementary Information. [file 41598_2024_52794_MOESM1_ESM.docx]

**Supplementary Material for “Light pollution disrupts circadian clock gene expression in two mosquito vectors during their overwintering dormancy”**

Figure S1. Daily mRNA expression profiles of core circadian clock genes in mature oocytes dissected from female *Aedes albopictus*. Gene expression in short-day reared (11.5:12.5 L:D, 20°C), diapausing control mosquitoes (ALAN-) are shown in blue triangles and dashed lines, while gene expression in those exposed to ALAN under the same conditions (ALAN+) are shown in orange circles and solid lines. Smaller markers represent relative mRNA abundance in biological replicates consisting of dissected oocytes from 8-10 females; and the large marker and bars represent mean ± standard error of 4-5 biological replicates. Spline curves were fit to the data using R. Photophase (white bar) and scotophase (colored bar) are represented in Zeitgeber (ZT) under the x-axis, with exposure to ALAN shown in the shaded orange bar.

Table S1. Primers for core circadian clock genes in *Aedes albopictus.*

| **Gene** | **Homologous Species** | **Primer Name** | **Primer Sequence (5’ - 3’)** | **Product Size** | **Melting Temp (°C)** |
| --- | --- | --- | --- | --- | --- |
| *timeless*  (JN59770.1) | N/A | Aalb_tim_qFw2 | GCGTCCTGTAATTCCGGGAA | 97 | 60.11 |
|  |  | Aalb_tim_qRev2 | TTTCCTTCAGCAAGCGGTCA |  | 60.18 |
| *period*  (JN559769.1) | N/A | Aalb_per_qFw1 | TTCAGTACGCGTCACTCCAC | 102 | 60.04 |
|  |  | Aalb_per_qRev1 | AATCGAACGGCCCAGAATGT |  | 60.04 |
| *cycle*  (XM_029879117.1) | *Aedes aegypti*  (JN573265.1) | Aalb_cyc_qFw1 | CAAACTGACGGTCCTTCGGA | 85 | 59.97 |
|  |  | Aalb_cyc_qRev1 | CTTGTAGTGGCCTTCGGTGT |  | 59.97 |
| *Clock*  (XM_019708616.2) | *Culex pipiens*  (KM355975.1) | Aalb_clk_qFw1 | CCGAAGAACAAGCGCAAACA | 80 | 59.97 |
|  |  | Aalb_clk_qRev1 | TTGCGATTGAGGAAGGTGCT |  | 59.96 |
| *cryptochrome1*  (XM_029880556.1) | *Culex pipiens*  (KM355976.1) | Aalb_cry1_qFw2 | GCACACCGTCAACATCATCG | 98 | 59.9 |
|  |  | Aalb_cry1_qRev2 | CCTGGGCAAGAATCGATGGA |  | 59.82 |
| *cryptochrome2*  (XM_019693841.2) | *Culex pipiens*  (KM355977.1) | Aalb_cry2_qFw1 | GCGTCTTGAGGAGCAGATGA | 89 | 59.83 |
|  |  | Aalb_cry2_qRev1 | TTGTGCTCGGTTCCAGTGAA |  | 59.82 |

Table S2. Calculated efficiency from standard curves for clock gene primers in *Aedes albopictus*.

| Gene Product | Efficiency | R^2^ Value | Number of points on Standard Curve |
| --- | --- | --- | --- |
| *timeless* | 103% | 0.9871 | 5 |
| *period* | 109% | 0.9814 | 5 |
| *cycle* | 98% | 0.9889 | 6 |
| *Clock* | 117% | 0.9910 | 6 |
| *cryptochrome1* | 118% | 0.9840 | 6 |
| *cryptochrome2* | 101% | 0.9839 | 5 |

Table S3. Summary statistics for two-way ANOVA evaluating suitability of reference genes (i.e., reference genes do not vary across daily time or between light treatments).

| **Reference Gene Group** | **Model Term** | **F-value** | **DF** | **P-value** |
| --- | --- | --- | --- | --- |
| *Cx. pipiens*  (*Rp49* & *RpL19*) | ZT | F = 1.626 | 5 | 0.171 |
|  | ALAN | F = 0.157 | 1 | 0.693 |
|  | ZTxALAN | F = 1.425 | 5 | 0.232 |
| Maternal *Ae. albopictus*  (*RpL34*, *RpL32*, & *RpS17*) | ZT | F = 0.535 | 5 | 0.748 |
|  | ALAN | F = 0.335 | 1 | 0.565 |
|  | ZTxALAN | F = 0.504 | 5 | 0.772 |
| *Ae. albopictus* oocytes  (*RpL32* & *RpL34*) | ZT | F = 0.484 | 5 | 0.786 |
|  | ALAN | F = 1.033 | 1 | 0.314 |
|  | ZTxALAN | F = 0.472 | 5 | 0.795 |

Table S4. Transformations and GLMs performed for each linear model. Residuals were checked for normality and tests for homogeneity of variances were used to assess suitability of the model. Response variables were transformed or a GLM used to ensure that models fit normality and homogeneity of variance assumptions. Where these were not needed (i.e. residuals were normal), no data transformations were applied.

| Species & Tissue | Gene | Model | Model Type |
| --- | --- | --- | --- |
| Adult females of *Cx. pipiens* | *cycle* | ALAN- Only | Logarithmic Transformation |
|  |  | ALAN+ Only | Logarithmic Transformation |
|  |  | ZT x Light Treatment | Logarithmic Transformation |
|  | *Clock* | ALAN- Only | No Transformation |
|  |  | ALAN+ Only | No Transformation |
|  |  | ZT x Light Treatment | No Transformation |
|  | *period* | ALAN- Only | Logarithmic Transformation |
|  |  | ALAN+ Only | GLM (Gamma, Log Link) |
|  |  | ZT x Light Treatment | Logarithmic Transformation |
|  | *timeless* | ALAN- Only | Logarithmic Transformation |
|  |  | ALAN+ Only | Logarithmic Transformation |
|  |  | ZT x Light Treatment | GLM (Inverse Gaussian, identity link) |
|  | *Pdp1* | ALAN- Only | Logarithmic Transformation |
|  |  | ALAN+ Only | Logarithmic Transformation |
|  |  | ZT x Light Treatment | Logarithmic Transformation |
|  | *cryptochrome2* | ALAN- Only | Logarithmic Transformation |
|  |  | ALAN+ Only | Logarithmic Transformation |
|  |  | ZT x Light Treatment | Logarithmic Transformation |
| Adult females of *Ae. albopictus* | *cycle* | ALAN- Only | Logarithmic Transformation |
|  |  | ALAN+ Only | Logarithmic Transformation |
|  |  | ZT x Light Treatment | Logarithmic Transformation |
|  | *Clock* | ALAN- Only | No Transformation |
|  |  | ALAN+ Only | Logarithmic Transformation |
|  |  | ZT x Light Treatment | Logarithmic Transformation |
|  | *period* | ALAN- Only | Logarithmic Transformation |
|  |  | ALAN+ Only | No Transformation |
|  |  | ZT x Light Treatment | Logarithmic Transformation |
|  | *timeless* | ALAN- Only | Logarithmic Transformation |
|  |  | ALAN+ Only | No Transformation |
|  |  | ZT x Light Treatment | GLM (Gamma, Log link) |
|  | *cryptochrome1* | ALAN- Only | Logarithmic Transformation |
|  |  | ALAN+ Only | Logarithmic Transformation |
|  |  | ZT x Light Treatment | Logarithmic Transformation |
|  | *cryptochrome2* | ALAN- Only | GLM (Gamma, Log Link) |
|  |  | ALAN+ Only | Logarithmic Transformation |
|  |  | ZT x Light Treatment | Logarithmic Transformation |
| Oocytes of *Ae. albopictus* | *cycle* | ALAN- Only | Logarithmic Transformation |
|  |  | ALAN+ Only | Logarithmic Transformation |
|  |  | ZT x Light Treatment | Logarithmic Transformation |
|  | *Clock* | ALAN- Only | Logarithmic Transformation |
|  |  | ALAN+ Only | Logarithmic Transformation |
|  |  | ZT x Light Treatment | Logarithmic Transformation |
|  | *period* | ALAN- Only | Logarithmic Transformation |
|  |  | ALAN+ Only | Logarithmic Transformation |
|  |  | ZT x Light Treatment | Logarithmic Transformation |
|  | *timeless* | ALAN- Only | No Transformation |
|  |  | ALAN+ Only | Logarithmic Transformation |
|  |  | ZT x Light Treatment | No Transformation |
|  | *cryptochrome1* | ALAN- Only | No Transformation |
|  |  | ALAN+ Only | Logarithmic Transformation |
|  |  | ZT x Light Treatment | Logarithmic Transformation |
|  | *cryptochrome2* | ALAN- Only | Logarithmic Transformation |
|  |  | ALAN+ Only | Logarithmic Transformation |
|  |  | ZT x Light Treatment | Logarithmic Transformation |

Table S5. Contrasts for time points (ZT) for all single-variable models of only ALAN- or ALAN+ within heads of adult females of *Cx. pipiens*. For all GLMs, contrasts are reported with a z ratio and for all LMs contrasts are reported with a t ratio. * indicates comparison used to determine daily cycling of transcripts.

| **Light Treatment** | **Gene** | **Contrast** | **t ratio**  **OR**  **z ratio** | **P value** |
| --- | --- | --- | --- | --- |
| ALAN- | *cycle* | ZT1 – ZT5 | 3.638 | **0.0146** |
|  |  | ZT1 – ZT9 | 17.497 | **< 0.0001** |
|  |  | ZT1 – ZT13 | 21.718 | **< 0.0001** |
|  |  | ZT1 – ZT17 | 13.227 | **< 0.0001** |
|  |  | ZT1 – ZT21 | 3.556 | **0.0177** |
|  |  | ZT5 – ZT9 | 13.859 | **< 0.0001** |
|  |  | ZT5 – ZT13 | 18.080 | **< 0.0001** |
|  |  | ZT5 – ZT17* | 9.589 | **< 0.0001** |
|  |  | ZT5 – ZT21 | -0.082 | 1.000 |
|  |  | ZT9 – ZT13 | 4.221 | **0.0036** |
|  |  | ZT9 – ZT17 | -4.271 | **0.0032** |
|  |  | ZT9 – ZT21 | -13.942 | **< 0.0001** |
|  |  | ZT13 – ZT17 | -8.492 | **< 0.0001** |
|  |  | ZT13 – ZT21 | -18.163 | **< 0.0001** |
|  |  | ZT17 – ZT21 | -9.671 | **<0.0001** |
| ALAN- | *Clock* | ZT1 – ZT5 | 1.347 | 0.7564 |
|  |  | ZT1 – ZT9 | 4.945 | **0.0006** |
|  |  | ZT1 – ZT13 | 3.430 | **0.0236** |
|  |  | ZT1 – ZT17 | -2.820 | 0.0883 |
|  |  | ZT1 – ZT21 | -2.548 | 0.1499 |
|  |  | ZT5 – ZT9 | 3.598 | **0.0161** |
|  |  | ZT5 – ZT13 | 2.083 | 0.3288 |
|  |  | ZT5 – ZT17* | -4.167 | **0.0041** |
|  |  | ZT5 – ZT21 | -3.895 | **0.0080** |
|  |  | ZT9 – ZT13 | -1.515 | 0.6584 |
|  |  | ZT9 – ZT17 | -7.765 | **< 0.0001** |
|  |  | ZT9 – ZT21 | -7.493 | **<0.0001** |
|  |  | ZT13 – ZT17 | -6.250 | **< 0.0001** |
|  |  | ZT13 – ZT21 | -5.978 | **< 0.0001** |
|  |  | ZT17 – ZT21 | 0.272 | 0.9998 |
| ALAN- | *period* | ZT1 – ZT5 | 9.483 | **< 0.0001** |
|  |  | ZT1 – ZT9 | -0.896 | 0.9434 |
|  |  | ZT1 – ZT13 | -8.509 | **< 0.0001** |
|  |  | ZT1 – ZT17 | -10.141 | **< 0.0001** |
|  |  | ZT1 – ZT21 | -4.465 | **0.0023** |
|  |  | ZT5 – ZT9 | -10.328 | **< 0.0001** |
|  |  | ZT5 – ZT13 | -17.505 | **< 0.0001** |
|  |  | ZT5 – ZT17* | -19.044 | **< 0.0001** |
|  |  | ZT5 – ZT21 | -13.232 | **< 0.0001** |
|  |  | ZT9 – ZT13 | -7.612 | **< 0.0001** |
|  |  | ZT9 – ZT17 | -9.245 | **< 0.0001** |
|  |  | ZT9 – ZT21 | -3.620 | **0.0166** |
|  |  | ZT13 – ZT17 | -1.632 | 0.5873 |
|  |  | ZT13 – ZT21 | 3.557 | **0.0191** |
|  |  | ZT17 – ZT21 | 5.096 | **0.0005** |
| ALAN- | *timeless* | ZT1 – ZT5 | 2.787 | 0.0943 |
|  |  | ZT1 – ZT9 | -5.169 | **0.0004** |
|  |  | ZT1 – ZT13 | -8.417 | **< 0.0001** |
|  |  | ZT1 – ZT17 | -9.459 | **< 0.0001** |
|  |  | ZT1 – ZT21 | -5.089 | **0.0004** |
|  |  | ZT5 – ZT9 | -7.956 | **< 0.0001** |
|  |  | ZT5 – ZT13 | -11.204 | **< 0.0001** |
|  |  | ZT5 – ZT17* | -12.247 | **< 0.0001** |
|  |  | ZT5 – ZT21 | -7.876 | **< 0.0001** |
|  |  | ZT9 – ZT13 | -3.247 | **0.0356** |
|  |  | ZT9 – ZT17 | -4.290 | **0.0031** |
|  |  | ZT9 – ZT21 | 0.081 | 1.000 |
|  |  | ZT13 – ZT17 | -1.043 | 0.8986 |
|  |  | ZT13 – ZT21 | 3.328 | **0.0298** |
|  |  | ZT17 – ZT21 | 4.371 | **0.0025** |
| ALAN- | *Pdp1* | ZT1 – ZT5 | -0.364 | 0.9990 |
|  |  | ZT1 – ZT9 | -5.335 | **0.0002** |
|  |  | ZT1 – ZT13 | -8.688 | **< 0.0001** |
|  |  | ZT1 – ZT17 | -9.698 | **< 0.0001** |
|  |  | ZT1 – ZT21 | -6.478 | **< 0.0001** |
|  |  | ZT5 – ZT9 | -4.971 | **0.0006** |
|  |  | ZT5 – ZT13 | -8.324 | **< 0.0001** |
|  |  | ZT5 – ZT17* | -9.334 | **< 0.0001** |
|  |  | ZT5 – ZT21 | -6.114 | **< 0.0001** |
|  |  | ZT9 – ZT13 | -3.353 | **0.0281** |
|  |  | ZT9 – ZT17 | -4.363 | **0.0026** |
|  |  | ZT9 – ZT21 | -1.142 | 0.8587 |
|  |  | ZT13 – ZT17 | -1.010 | 0.9102 |
|  |  | ZT13 – ZT21 | 2.211 | 0.2695 |
|  |  | ZT17 – ZT21 | 3.220 | **0.0378** |
| ALAN- | *cryptochrome2* | ZT1 – ZT5 | 12.054 | **< 0.0001** |
|  |  | ZT1 – ZT9 | 14.641 | **< 0.0001** |
|  |  | ZT1 – ZT13 | -6.217 | **< 0.0001** |
|  |  | ZT1 – ZT17 | -13.542 | **< 0.0001** |
|  |  | ZT1 – ZT21 | -7.274 | **< 0.0001** |
|  |  | ZT5 – ZT9 | 2.587 | 0.1394 |
|  |  | ZT5 – ZT13 | -18.272 | **< 0.0001** |
|  |  | ZT5 – ZT17* | -25.597 | **< 0.0001** |
|  |  | ZT5 – ZT21 | -19.329 | **< 0.0001** |
|  |  | ZT9 – ZT13 | -20.858 | **< 0.0001** |
|  |  | ZT9 – ZT17 | -28.183 | **< 0.0001** |
|  |  | ZT9 – ZT21 | -21.915 | **< 0.0001** |
|  |  | ZT13 – ZT17 | -7.325 | **< 0.0001** |
|  |  | ZT13 – ZT21 | -1.057 | 0.8932 |
|  |  | ZT17 – ZT21 | 6.268 | **< 0.0001** |
| ALAN+ | *cycle* | ZT1 – ZT5 | 2.820 | 0.0882 |
|  |  | ZT1 – ZT9 | 15.368 | **< 0.0001** |
|  |  | ZT1 – ZT13 | 14.363 | **< 0.0001** |
|  |  | ZT1 – ZT17 | 10.365 | **< 0.0001** |
|  |  | ZT1 – ZT21 | 3.145 | **0.0446** |
|  |  | ZT5 – ZT9 | 12.548 | **< 0.0001** |
|  |  | ZT5 – ZT13 | 11.543 | **< 0.0001** |
|  |  | ZT5 – ZT17* | 7.545 | **< 0.0001** |
|  |  | ZT5 – ZT21 | 0.324 | 0.995 |
|  |  | ZT9 – ZT13 | -1.005 | 0.9117 |
|  |  | ZT9 – ZT17 | -5.003 | **0.0005** |
|  |  | ZT9 – ZT21 | -12.224 | **< 0.0001** |
|  |  | ZT13 – ZT17 | -3.998 | **0.0062** |
|  |  | ZT13 – ZT21 | -11.219 | **< 0.0001** |
|  |  | ZT17 – ZT21 | -7.221 | **< 0.0001** |
| ALAN+ | *Clock* | ZT1 – ZT5 | 0.851 | 0.9544 |
|  |  | ZT1 – ZT9 | 4.636 | **0.0013** |
|  |  | ZT1 – ZT13 | 2777 | 0.0962 |
|  |  | ZT1 – ZT17 | 1.248 | 0.8093 |
|  |  | ZT1 – ZT21 | -1.683 | 0.5555 |
|  |  | ZT5 – ZT9 | 3.784 | **0.0104** |
|  |  | ZT5 – ZT13 | 1.926 | 0.4114 |
|  |  | ZT5 – ZT17* | 0.397 | 0.9986 |
|  |  | ZT5 – ZT21 | -2.534 | 0.1539 |
|  |  | ZT9 – ZT13 | -1.858 | 0.4501 |
|  |  | ZT9 – ZT17 | -3.388 | **0.0260** |
|  |  | ZT9 – ZT21 | -6.318 | **< 0.0001** |
|  |  | ZT13 – ZT17 | -1.530 | 0.6494 |
|  |  | ZT13 – ZT21 | -4.460 | **0.0020** |
|  |  | ZT17 – ZT21 | -2.930 | 0.0704 |
| ALAN+ | *period* | ZT1 – ZT5 | 7.253 | **< 0.0001** |
|  |  | ZT1 – ZT9 | 0.894 | 0.9480 |
|  |  | ZT1 – ZT13 | -3.133 | **0.0214** |
|  |  | ZT1 – ZT17 | -4.175 | **0.0004** |
|  |  | ZT1 – ZT21 | -0.920 | 0.9415 |
|  |  | ZT5 – ZT9 | -6.358 | **< 0.0001** |
|  |  | ZT5 – ZT13 | -9.971 | **< 0.0001** |
|  |  | ZT5 – ZT17* | -11.428 | **< 0.0001** |
|  |  | ZT5 – ZT21 | -8.173 | **< 0.0001** |
|  |  | ZT9 – ZT13 | -3.976 | **0.0010** |
|  |  | ZT9 – ZT17 | -5.069 | **< 0.0001** |
|  |  | ZT9 – ZT21 | -1.814 | 0.4563 |
|  |  | ZT13 – ZT17 | -0.804 | 0.9670 |
|  |  | ZT13 – ZT21 | 2.265 | 0.2084 |
|  |  | ZT17 – ZT21 | 3.255 | **0.0144** |
| ALAN+ | *timeless* | ZT1 – ZT5 | 0.429 | 0.9979 |
|  |  | ZT1 – ZT9 | -2.362 | 0.2092 |
|  |  | ZT1 – ZT13 | **-3.691** | **0.0129** |
|  |  | ZT1 – ZT17 | **-5.098** | **0.0004** |
|  |  | ZT1 – ZT21 | -2.580 | 0.1411 |
|  |  | ZT5 – ZT9 | **-2.792** | **0.0935** |
|  |  | ZT5 – ZT13 | **-4.120** | **0.0046** |
|  |  | ZT5 – ZT17* | **-5.528** | **0.0001** |
|  |  | ZT5 – ZT21 | -3.009 | 0.0596 |
|  |  | ZT9 – ZT13 | -1.329 | 0.7667 |
|  |  | ZT9 – ZT17 | -2.736 | 0.1044 |
|  |  | ZT9 – ZT21 | -0.218 | 0.9999 |
|  |  | ZT13 – ZT17 | -1.407 | 0.7225 |
|  |  | ZT13 – ZT21 | 1.111 | 0.8721 |
|  |  | ZT17 – ZT21 | 2.518 | 0.1583 |
| ALAN+ | *Pdp1* | ZT1 – ZT5 | -0.368 | 0.9990 |
|  |  | ZT1 – ZT9 | -3.701 | **0.0126** |
|  |  | ZT1 – ZT13 | -6.270 | **< 0.0001** |
|  |  | ZT1 – ZT17 | -5.747 | **0.0001** |
|  |  | ZT1 – ZT21 | -4.112 | **0.0047** |
|  |  | ZT5 – ZT9 | -3.333 | **0.0294** |
|  |  | ZT5 – ZT13 | -5.902 | **0.0001** |
|  |  | ZT5 – ZT17* | -5.380 | **0.0002** |
|  |  | ZT5 – ZT21 | -3.744 | **0.0114** |
|  |  | ZT9 – ZT13 | -2.569 | **0.1441** |
|  |  | ZT9 – ZT17 | -2.046 | 0.3472 |
|  |  | ZT9 – ZT21 | -0.411 | 0.9983 |
|  |  | ZT13 – ZT17 | 0.523 | 0.9947 |
|  |  | ZT13 – ZT21 | 2.158 | 0.2930 |
|  |  | ZT17 – ZT21 | 1.635 | 0.5845 |
| ALAN+ | *cryptochrome2* | ZT1 – ZT5 | **5.047** | **0.0005** |
|  |  | ZT1 – ZT9 | **4.806** | **0.0009** |
|  |  | ZT1 – ZT13 | **-4.555** | **0.0016** |
|  |  | ZT1 – ZT17 | **-5.974** | **< 0.0001** |
|  |  | ZT1 – ZT21 | **-4.129** | **0.0045** |
|  |  | ZT5 – ZT9 | -0.241 | 0.9999 |
|  |  | ZT5 – ZT13 | **-9.602** | **< 0.0001** |
|  |  | ZT5 – ZT17* | **-11.021** | **< 0.0001** |
|  |  | ZT5 – ZT21 | **-9.176** | **< 0.0001** |
|  |  | ZT9 – ZT13 | **-9.361** | **< 0.0001** |
|  |  | ZT9 – ZT17 | **-10.780** | **< 0.0001** |
|  |  | ZT9 – ZT21 | **-8.935** | **< 0.0001** |
|  |  | ZT13 – ZT17 | -1.419 | 0.7156 |
|  |  | ZT13 – ZT21 | 0.426 | 0.9980 |
|  |  | ZT17 – ZT21 | 1.845 | 0.4578 |

Table S6. Contrasts for time points (ZT) for all single-variable models of only ALAN- or ALAN+ within heads of adult females of *Ae. albopictus*. For all GLMs, contrasts are reported with a z ratio and for all LMs contrasts are reported with a t ratio. * indicates comparison used to determine daily cycling of transcripts.

| **Light Treatment** | **Gene** | **Contrast** | **t ratio**  **OR**  **z ratio** | **P value** |
| --- | --- | --- | --- | --- |
| ALAN- | *cycle* | ZT1 – ZT5 | 1.535 | 0.6464 |
|  |  | ZT1 – ZT9 | 2.283 | 0.2427 |
|  |  | ZT1 – ZT13 | 7.601 | **< 0.0001** |
|  |  | ZT1 – ZT17 | 6.148 | **< 0.0001** |
|  |  | ZT1 – ZT21 | 4.044 | **0.0063** |
|  |  | ZT5 – ZT9 | 0.793 | 0.9659 |
|  |  | ZT5 – ZT13 | 6.477 | **< 0.0001** |
|  |  | ZT5 – ZT17* | 4.892 | **0.0009** |
|  |  | ZT5 – ZT21 | 2.661 | 0.1242 |
|  |  | ZT9 – ZT13 | 5.730 | **0.0001** |
|  |  | ZT9 – ZT17 | 4.100 | **0.0055** |
|  |  | ZT9 – ZT21 | 1.868 | 0.4460 |
|  |  | ZT13 – ZT17 | -18.868 | 0.4482 |
|  |  | ZT13 – ZT21 | -3.968 | **0.0075** |
|  |  | ZT17 – ZT21 | -2.231 | 0.2637 |
| ALAN- | *Clock* | ZT1 – ZT5 | 1.629 | 0.5894 |
|  |  | ZT1 – ZT9 | 4.698 | **0.0014** |
|  |  | ZT1 – ZT13 | 5.871 | **0.0001** |
|  |  | ZT1 – ZT17 | 4.974 | **0.0007** |
|  |  | ZT1 – ZT21 | 4.383 | **0.0028** |
|  |  | ZT5 – ZT9 | 3.255 | **0.0371** |
|  |  | ZT5 – ZT13 | 4.560 | **0.0019** |
|  |  | ZT5 – ZT17* | 3.548 | **0.0195** |
|  |  | ZT5 – ZT21 | 2.921 | **0.0746** |
|  |  | ZT9 – ZT13 | 1.490 | 0.6736 |
|  |  | ZT9 – ZT17 | 0.293 | 0.9997 |
|  |  | ZT9 – ZT21 | -0.334 | 0.9994 |
|  |  | ZT13 – ZT17 | -1.214 | 0.8254 |
|  |  | ZT13 – ZT21 | -1.805 | 0.4828 |
|  |  | ZT17 – ZT21 | -0.627 | 0.9877 |
| ALAN- | *period* | ZT1 – ZT5 | 1.183 | 0.8401 |
|  |  | ZT1 – ZT9 | -2.637 | 0.1299 |
|  |  | ZT1 – ZT13 | -3.389 | **0.0278** |
|  |  | ZT1 – ZT17 | -5.855 | **0.0001** |
|  |  | ZT1 – ZT21 | -0.750 | 0.9730 |
|  |  | ZT5 – ZT9 | -4.052 | **0.0062** |
|  |  | ZT5 – ZT13 | -4.755 | **0.0012** |
|  |  | ZT5 – ZT17* | -7.465 | **< 0.0001** |
|  |  | ZT5 – ZT21 | -2.050 | 0.3478 |
|  |  | ZT9 – ZT13 | -0.935 | 0.9331 |
|  |  | ZT9 – ZT17 | -3.413 | **0.0253** |
|  |  | ZT9 – ZT21 | 2.002 | 0.3726 |
|  |  | ZT13 – ZT17 | -2.283 | 0.2425 |
|  |  | ZT13 – ZT21 | 2.822 | 0.0910 |
|  |  | ZT17 – ZT21 | 5.415 | **0.0002** |
| ALAN- | *timeless* | ZT1 – ZT5 | 0.026 | 1.000 |
|  |  | ZT1 – ZT9 | 0.217 | 0.9999 |
|  |  | ZT1 – ZT13 | -0.381 | 0.9988 |
|  |  | ZT1 – ZT17 | -0.525 | 0.9946 |
|  |  | ZT1 – ZT21 | 3.068 | 0.0551 |
|  |  | ZT5 – ZT9 | 0.202 | 0.9999 |
|  |  | ZT5 – ZT13 | -0.428 | 0.9979 |
|  |  | ZT5 – ZT17* | -0.584 | 0.9911 |
|  |  | ZT5 – ZT21 | 3.226 | **0.0395** |
|  |  | ZT9 – ZT13 | -0.619 | 0.9884 |
|  |  | ZT9 – ZT17 | -0.787 | 0.9669 |
|  |  | ZT9 – ZT21 | 3.024 | 0.0604 |
|  |  | ZT13 – ZT17 | -0.123 | 1.000 |
|  |  | ZT13 – ZT21 | 3.470 | **0.0232** |
|  |  | ZT17 – ZT21 | 3.811 | **0.0108** |
| ALAN- | *cryptochrome1* | ZT1 – ZT5 | 2.125 | 0.3113 |
|  |  | ZT1 – ZT9 | 0.724 | 0.9768 |
|  |  | ZT1 – ZT13 | 2.543 | 0.1545 |
|  |  | ZT1 – ZT17 | 2.849 | 0.0861 |
|  |  | ZT1 – ZT21 | 3.873 | **0.0093** |
|  |  | ZT5 – ZT9 | -1.486 | 0.6764 |
|  |  | ZT5 – ZT13 | 0.556 | 0.9929 |
|  |  | ZT5 – ZT17* | 0.769 | 0.9700 |
|  |  | ZT5 – ZT21 | 1.854 | 0.4542 |
|  |  | ZT9 – ZT13 | 1.957 | 0.3967 |
|  |  | ZT9 – ZT17 | 2.254 | 0.2542 |
|  |  | ZT9 – ZT21 | 3.340 | **0.0309** |
|  |  | ZT13 – ZT17 | 0.168 | 1.000 |
|  |  | ZT13 – ZT21 | 1.192 | 0.8359 |
|  |  | ZT17 – ZT21 | 1.086 | 0.8819 |
| ALAN- | *cryptochrome2* | ZT1 – ZT5 | 1.000 | 0.9181 |
|  |  | ZT1 – ZT9 | 2.023 | 0.3292 |
|  |  | ZT1 – ZT13 | 2.585 | 0.1008 |
|  |  | ZT1 – ZT17 | 2.001 | 0.3416 |
|  |  | ZT1 – ZT21 | 2.335 | 0.1801 |
|  |  | ZT5 – ZT9 | 1.085 | 0.8877 |
|  |  | ZT5 – ZT13 | 1.725 | 0.5150 |
|  |  | ZT5 – ZT17* | 1.062 | 0.8965 |
|  |  | ZT5 – ZT21 | 1.416 | 0.7175 |
|  |  | ZT9 – ZT13 | 0.703 | 0.9817 |
|  |  | ZT9 – ZT17 | -0.023 | 1.000 |
|  |  | ZT9 – ZT21 | 0.331 | 0.9995 |
|  |  | ZT13 – ZT17 | -0.724 | 0.9791 |
|  |  | ZT13 – ZT21 | -0.390 | 0.9988 |
|  |  | ZT17 – ZT21 | 0.354 | 0.9993 |
| ALAN+ | *cycle* | ZT1 – ZT5 | 2.606 | 0.1360 |
|  |  | ZT1 – ZT9 | 2.633 | 0.1293 |
|  |  | ZT1 – ZT13 | 3.043 | 0.0567 |
|  |  | ZT1 – ZT17 | 2.792 | 0.0949 |
|  |  | ZT1 – ZT21 | 3.678 | **0.0139** |
|  |  | ZT5 – ZT9 | 0.029 | 1.000 |
|  |  | ZT5 – ZT13 | 0.464 | 0.9969 |
|  |  | ZT5 – ZT17* | 0.198 | 1.000 |
|  |  | ZT5 – ZT21 | 1.137 | 0.8609 |
|  |  | ZT9 – ZT13 | 0.436 | 0.9977 |
|  |  | ZT9 – ZT17 | 0.169 | 1.000 |
|  |  | ZT9 – ZT21 | 1.108 | 0.8729 |
|  |  | ZT13 – ZT17 | -0.266 | 0.9998 |
|  |  | ZT13 – ZT21 | 0.673 | 0.9833 |
|  |  | ZT17 – ZT21 | 0.939 | 0.9320 |
| ALAN+ | *Clock* | ZT1 – ZT5 | 4.216 | **0.0039** |
|  |  | ZT1 – ZT9 | 3.796 | **0.0106** |
|  |  | ZT1 – ZT13 | 3.796 | **0.0147** |
|  |  | ZT1 – ZT17 | 4.028 | **0.0061** |
|  |  | ZT1 – ZT21 | 5.723 | **0.0001** |
|  |  | ZT5 – ZT9 | -0.445 | 0.9975 |
|  |  | ZT5 – ZT13 | -0.596 | 0.9903 |
|  |  | ZT5 – ZT17* | -0.199 | 1.000 |
|  |  | ZT5 – ZT21 | 1.599 | 0.6073 |
|  |  | ZT9 – ZT13 | -0.151 | 1.000 |
|  |  | ZT9 – ZT17 | 0.246 | 0.9999 |
|  |  | ZT9 – ZT21 | 2.044 | 0.3496 |
|  |  | ZT13 – ZT17 | 0.397 | 0.9986 |
|  |  | ZT13 – ZT21 | 2.195 | 0.2780 |
|  |  | ZT17 – ZT21 | 1.798 | 0.4862 |
| ALAN+ | *period* | ZT1 – ZT5 | 7.253 | **<0.0001** |
|  |  | ZT1 – ZT9 | 0.894 | 0.9480 |
|  |  | ZT1 – ZT13 | -3.133 | **0.0214** |
|  |  | ZT1 – ZT17 | -4.175 | **0.0004** |
|  |  | ZT1 – ZT21 | -0.920 | 0.9415 |
|  |  | ZT5 – ZT9 | -6.358 | **<0.0001** |
|  |  | ZT5 – ZT13 | -9.971 | **<0.0001** |
|  |  | ZT5 – ZT17* | -11.428 | **<0.0001** |
|  |  | ZT5 – ZT21 | -8.173 | **<0.0001** |
|  |  | ZT9 – ZT13 | -3.976 | **0.0010** |
|  |  | ZT9 – ZT17 | -5.059 | **<0.0001** |
|  |  | ZT9 – ZT21 | -1.814 | 0.4563 |
|  |  | ZT13 – ZT17 | -0.804 | 0.9670 |
|  |  | ZT13 – ZT21 | 2.265 | 0.2084 |
|  |  | ZT17 – ZT21 | 3.255 | **0.0144** |
| ALAN+ | *timeless* | ZT1 – ZT5 | 3.193 | **0.0412** |
|  |  | ZT1 – ZT9 | 1.200 | 0.8325 |
|  |  | ZT1 – ZT13 | 0.847 | 0.9552 |
|  |  | ZT1 – ZT17 | 0.450 | 0.9974 |
|  |  | ZT1 – ZT21 | 2.807 | 0.0921 |
|  |  | ZT5 – ZT9 | -2.114 | 0.3150 |
|  |  | ZT5 – ZT13 | -2.488 | 0.1689 |
|  |  | ZT5 – ZT17* | -2.910 | 0.0749 |
|  |  | ZT5 – ZT21 | -0.409 | 0.9983 |
|  |  | ZT9 – ZT13 | -0.374 | 0.9989 |
|  |  | ZT9 – ZT17 | -0.795 | 0.9655 |
|  |  | ZT9 – ZT21 | 1.705 | 0.5422 |
|  |  | ZT13 – ZT17 | -0.421 | 0.9981 |
|  |  | ZT13 – ZT21 | 2.079 | 0.3320 |
|  |  | ZT17 – ZT21 | 2.500 | 0.1652 |
| ALAN+ | *cryptochrome1* | ZT1 – ZT5 | 5.158 | **0.0004** |
|  |  | ZT1 – ZT9 | 1.422 | 0.7137 |
|  |  | ZT1 – ZT13 | 1.810 | 0.4790 |
|  |  | ZT1 – ZT17 | 1.105 | 0.8745 |
|  |  | ZT1 – ZT21 | 2.834 | 0.0872 |
|  |  | ZT5 – ZT9 | -3.962 | **0.0072** |
|  |  | ZT5 – ZT13 | -3.551 | **0.0186** |
|  |  | ZT5 – ZT17* | -4.299 | **0.0032** |
|  |  | ZT5 – ZT21 | -2.465 | 0.1762 |
|  |  | ZT9 – ZT13 | 0.411 | 0.9983 |
|  |  | ZT9 – ZT17 | -0.337 | 0.9993 |
|  |  | ZT9 – ZT21 | 1.498 | 0.6690 |
|  |  | ZT13 – ZT17 | -0.748 | 0.9734 |
|  |  | ZT13 – ZT21 | 1.086 | 0.8818 |
|  |  | ZT17 – ZT21 | 1.835 | 0.4645 |
| ALAN+ | *cryptochrome2* | ZT1 – ZT5 | 3.172 | **0.0431** |
|  |  | ZT1 – ZT9 | 2.790 | 0.0953 |
|  |  | ZT1 – ZT13 | 1.613 | 0.5987 |
|  |  | ZT1 – ZT17 | 1.889 | 0.4335 |
|  |  | ZT1 – ZT21 | 4.826 | **0.0009** |
|  |  | ZT5 – ZT9 | -0.406 | 0.9984 |
|  |  | ZT5 – ZT13 | -1.654 | 0.5735 |
|  |  | ZT5 – ZT17* | -1.362 | 0.7484 |
|  |  | ZT5 – ZT21 | 1.754 | 0.5125 |
|  |  | ZT9 – ZT13 | -1.248 | 0.8089 |
|  |  | ZT9 – ZT17 | -0.955 | 0.9272 |
|  |  | ZT9 – ZT21 | 2.160 | 0.2935 |
|  |  | ZT13 – ZT17 | 0.292 | 0.9997 |
|  |  | ZT13 – ZT21 | 3.408 | **0.0257** |
|  |  | ZT17 – ZT21 | 3.116 | **0.0487** |

Table S7. Contrasts for time points (ZT) for all single-variable models of only ALAN- or ALAN+ within oocytes of *Ae.albopictus*. For all GLMs, contrasts are reported with a z ratio and for all LMs contrasts are reported with a t ratio. * indicates comparison used to determine daily cycling of transcripts.

| **Light Treatment** | **Gene** | **Contrast** | **t ratio**  **OR**  **z ratio** | **P value** |
| --- | --- | --- | --- | --- |
| ALAN- | *cycle* | ZT1 – ZT5 | 0.791 | 0.9664 |
|  |  | ZT1 – ZT9 | 0.474 | 0.9967 |
|  |  | ZT1 – ZT13 | 1.604 | 0.6041 |
|  |  | ZT1 – ZT17 | 2.356 | 0.2113 |
|  |  | ZT1 – ZT21 | 1.786 | 0.4928 |
|  |  | ZT5 – ZT9 | -0.318 | 0.9995 |
|  |  | ZT5 – ZT13 | 0.812 | 0.9624 |
|  |  | ZT5 – ZT17* | 1.565 | 0.6277 |
|  |  | ZT5 – ZT21 | 0.995 | 0.9152 |
|  |  | ZT9 – ZT13 | 1.130 | 0.8641 |
|  |  | ZT9 – ZT17 | 1.883 | 0.4359 |
|  |  | ZT9 – ZT21 | 1.312 | 0.7757 |
|  |  | ZT13 – ZT17 | 0.753 | 0.9728 |
|  |  | ZT13 – ZT21 | 0.182 | 1.000 |
|  |  | ZT17 – ZT21 | -0.571 | 0.9921 |
| ALAN- | *Clock* | ZT1 – ZT5 | 0.349 | 0.9993 |
|  |  | ZT1 – ZT9 | -0.462 | 0.9972 |
|  |  | ZT1 – ZT13 | 2.779 | 0.0774 |
|  |  | ZT1 – ZT17 | 2.974 | **0.0484** |
|  |  | ZT1 – ZT21 | 2.577 | 0.1217 |
|  |  | ZT5 – ZT9 | -0.781 | 0.9695 |
|  |  | ZT5 – ZT13 | 2355 | 0.1917 |
|  |  | ZT5 – ZT17* | 2.545 | 0.1304 |
|  |  | ZT5 – ZT21 | 2.159 | 0.2748 |
|  |  | ZT9 – ZT13 | 3.082 | **0.0368** |
|  |  | ZT9 – ZT17 | 3.266 | **0.0227** |
|  |  | ZT9 – ZT21 | 2.892 | 0.0592 |
|  |  | ZT13 – ZT17 | 0.195 | 1.0000 |
|  |  | ZT13 – ZT21 | -0.202 | 1.0000 |
|  |  | ZT17 – ZT21 | -0.397 | 0.9987 |
| ALAN- | *period* | ZT1 – ZT5 | 1.884 | 0.4362 |
|  |  | ZT1 – ZT9 | -1.427 | 0.7108 |
|  |  | ZT1 – ZT13 | 2.817 | 0.0902 |
|  |  | ZT1 – ZT17 | 1.685 | 0.5543 |
|  |  | ZT1 – ZT21 | 2.739 | 0.1053 |
|  |  | ZT5 – ZT9 | -3.203 | 0.0403 |
|  |  | ZT5 – ZT13 | 0.933 | 0.9336 |
|  |  | ZT5 – ZT17* | 1.199 | 1.0000 |
|  |  | ZT5 – ZT21 | 0.855 | 0.9533 |
|  |  | ZT9 – ZT13 | 4.083 | **0.0054** |
|  |  | ZT9 – ZT17 | 3.016 | 0.0600 |
|  |  | ZT9 – ZT21 | 4.010 | **0.0064** |
|  |  | ZT13 – ZT17 | -1.132 | 0.8630 |
|  |  | ZT13 – ZT21 | -0.078 | 1.0000 |
|  |  | ZT17 – ZT21 | 1.054 | 0.8943 |
| ALAN- | *timeless* | ZT1 – ZT5 | 1.977 | 0.3838 |
|  |  | ZT1 – ZT9 | 0.199 | 1.0000 |
|  |  | ZT1 – ZT13 | 0.850 | 0.9547 |
|  |  | ZT1 – ZT17 | 1.818 | 0.4736 |
|  |  | ZT1 – ZT21 | 0.628 | 0.9878 |
|  |  | ZT5 – ZT9 | -1.778 | 0.4975 |
|  |  | ZT5 – ZT13 | -1.127 | 0.8653 |
|  |  | ZT5 – ZT17* | -0.158 | 1.000 |
|  |  | ZT5 – ZT21 | -1.349 | 0.7555 |
|  |  | ZT9 – ZT13 | 0.651 | 0.9856 |
|  |  | ZT9 – ZT17 | 1.620 | 0.5943 |
|  |  | ZT9 – ZT21 | 0.429 | 0.9979 |
|  |  | ZT13 – ZT17 | 0.969 | 0.9234 |
|  |  | ZT13 – ZT21 | -0.222 | 0.9999 |
|  |  | ZT17 – ZT21 | -1.191 | 0.8370 |
| ALAN- | *cryptochrome1* | ZT1 – ZT5 | -0.971 | 0.9220 |
|  |  | ZT1 – ZT9 | -0.414 | 0.9982 |
|  |  | ZT1 – ZT13 | 0.271 | 0.9998 |
|  |  | ZT1 – ZT17 | 0.431 | 0.9979 |
|  |  | ZT1 – ZT21 | 1.761 | 0.5075 |
|  |  | ZT5 – ZT9 | 0.559 | 0.9928 |
|  |  | ZT5 – ZT13 | 1.245 | 0.8107 |
|  |  | ZT5 – ZT17* | 1.404 | 0.7244 |
|  |  | ZT5 – ZT21 | 2.735 | 0.1047 |
|  |  | ZT9 – ZT13 | 0.686 | 0.9819 |
|  |  | ZT9 – ZT17 | 0.845 | 0.9558 |
|  |  | ZT9 – ZT21 | 2.175 | 0.2851 |
|  |  | ZT13 – ZT17 | 0.159 | 1.000 |
|  |  | ZT13 – ZT21 | 1.490 | 0.6736 |
|  |  | ZT17 – ZT21 | 1.331 | 0.7656 |
| ALAN- | *cryptochrome2* | ZT1 – ZT5 | -0.496 | 0.9959 |
|  |  | ZT1 – ZT9 | -0.885 | 0.9465 |
|  |  | ZT1 – ZT13 | 0.820 | 0.9610 |
|  |  | ZT1 – ZT17 | 1.074 | 0.8868 |
|  |  | ZT1 – ZT21 | 1.529 | 0.6501 |
|  |  | ZT5 – ZT9 | -0.389 | 0.9987 |
|  |  | ZT5 – ZT13 | 1.316 | 0.7737 |
|  |  | ZT5 – ZT17* | 1.570 | 0.6247 |
|  |  | ZT5 – ZT21 | 2.025 | 0.3583 |
|  |  | ZT9 – ZT13 | 1.705 | 0.5419 |
|  |  | ZT9 – ZT17 | 1.959 | 0.3931 |
|  |  | ZT9 – ZT21 | 2.414 | 0.1911 |
|  |  | ZT13 – ZT17 | 0.255 | 0.9998 |
|  |  | ZT13 – ZT21 | 0.709 | 0.9790 |
|  |  | ZT17 – ZT21 | 0.455 | 0.9973 |
| ALAN+ | *cycle* | ZT1 – ZT5 | 1.994 | 0.3746 |
|  |  | ZT1 – ZT9 | -0.265 | 0.9998 |
|  |  | ZT1 – ZT13 | 1.522 | 0.6543 |
|  |  | ZT1 – ZT17 | 0.858 | 0.9528 |
|  |  | ZT1 – ZT21 | 0.080 | 1.0000 |
|  |  | ZT5 – ZT9 | -2.259 | 0.2491 |
|  |  | ZT5 – ZT13 | -0.472 | 0.9967 |
|  |  | ZT5 – ZT17* | -1.135 | 0.8618 |
|  |  | ZT5 – ZT21 | -1.913 | 0.4186 |
|  |  | ZT9 – ZT13 | 1.787 | 0.4919 |
|  |  | ZT9 – ZT17 | 1.124 | 0.8667 |
|  |  | ZT9 – ZT21 | 0.346 | 0.9993 |
|  |  | ZT13 – ZT17 | -0.664 | 0.9843 |
|  |  | ZT13 – ZT21 | -1.442 | 0.7024 |
|  |  | ZT17 – ZT21 | -0.778 | 0.9687 |
| ALAN+ | *Clock* | ZT1 – ZT5 | 0.933 | 0.9337 |
|  |  | ZT1 – ZT9 | -0.015 | 1.0000 |
|  |  | ZT1 – ZT13 | 1.612 | 0.5995 |
|  |  | ZT1 – ZT17 | 2.249 | 0.2549 |
|  |  | ZT1 – ZT21 | 2.729 | 0.1074 |
|  |  | ZT5 – ZT9 | -0.947 | 0.9297 |
|  |  | ZT5 – ZT13 | 0.586 | 0.9910 |
|  |  | ZT5 – ZT17* | 1.187 | 0.8385 |
|  |  | ZT5 – ZT21 | 1.640 | 0.5822 |
|  |  | ZT9 – ZT13 | 1.626 | 0.5904 |
|  |  | ZT9 – ZT17 | 2.263 | 0.2488 |
|  |  | ZT9 – ZT21 | 2.744 | 0.1043 |
|  |  | ZT13 – ZT17 | 0.637 | 0.9869 |
|  |  | ZT13 – ZT21 | 1.117 | 0.8692 |
|  |  | ZT17 – ZT21 | 0.480 | 0.9964 |
| ALAN+ | *period* | ZT1 – ZT5 | 0.980 | 0.9198 |
|  |  | ZT1 – ZT9 | -0.642 | 0.9855 |
|  |  | ZT1 – ZT13 | 0.801 | 0.9646 |
|  |  | ZT1 – ZT17 | 0.256 | 0.9998 |
|  |  | ZT1 – ZT21 | 0.549 | 0.9934 |
|  |  | ZT5 – ZT9 | -1.632 | 0.5866 |
|  |  | ZT5 – ZT13 | -0.179 | 1.0000 |
|  |  | ZT5 – ZT17* | -0.724 | 0.9770 |
|  |  | ZT5 – ZT21 | -0.432 | 0.9978 |
|  |  | ZT9 – ZT13 | 1.453 | 0.6958 |
|  |  | ZT9 – ZT17 | 0.908 | 0.9407 |
|  |  | ZT9 – ZT21 | 1.200 | 0.8324 |
|  |  | ZT13 – ZT17 | -0.545 | 0.9936 |
|  |  | ZT13 – ZT21 | -0.252 | 0.9998 |
|  |  | ZT17 – ZT21 | 0.292 | 0.9997 |
| ALAN+ | *timeless* | ZT1 – ZT5 | 1.789 | 0.4910 |
|  |  | ZT1 – ZT9 | -0.540 | 0.9939 |
|  |  | ZT1 – ZT13 | 1.211 | 0.8272 |
|  |  | ZT1 – ZT17 | 0.482 | 0.9964 |
|  |  | ZT1 – ZT21 | 0.501 | 0.9957 |
|  |  | ZT5 – ZT9 | -2.328 | 0.2217 |
|  |  | ZT5 – ZT13 | -0.578 | 0.9916 |
|  |  | ZT5 – ZT17* | -1.307 | 0.7785 |
|  |  | ZT5 – ZT21 | -1.288 | 0.7883 |
|  |  | ZT9 – ZT13 | 1.751 | 0.5139 |
|  |  | ZT9 – ZT17 | 1.022 | 0.9061 |
|  |  | ZT9 – ZT21 | 1.040 | 0.8995 |
|  |  | ZT13 – ZT17 | -0.729 | 0.9763 |
|  |  | ZT13 – ZT21 | -0.711 | 0.9788 |
|  |  | ZT17 – ZT21 | 0.018 | 1.0000 |
| ALAN+ | *cryptochrome1* | ZT1 – ZT5 | -2.304 | 0.2313 |
|  |  | ZT1 – ZT9 | -1.411 | 0.7202 |
|  |  | ZT1 – ZT13 | -0.082 | 1.0000 |
|  |  | ZT1 – ZT17 | -0.213 | 0.9999 |
|  |  | ZT1 – ZT21 | -0.854 | 0.9538 |
|  |  | ZT5 – ZT9 | 0.892 | 0.9446 |
|  |  | ZT5 – ZT13 | 2.222 | 0.2648 |
|  |  | ZT5 – ZT17* | 2.090 | 0.3251 |
|  |  | ZT5 – ZT21 | 1.450 | 0.6976 |
|  |  | ZT9 – ZT13 | 1.329 | 0.7665 |
|  |  | ZT9 – ZT17 | 1.198 | 0.8335 |
|  |  | ZT9 – ZT21 | 0.557 | 0.9929 |
|  |  | ZT13 – ZT17 | -0.131 | 1.0000 |
|  |  | ZT13 – ZT21 | -0.772 | 0.9697 |
|  |  | ZT17 – ZT21 | -0.641 | 0.9866 |
| ALAN+ | *cryptochrome2* | ZT1 – ZT5 | -0.993 | 0.9158 |
|  |  | ZT1 – ZT9 | -2.261 | 0.2481 |
|  |  | ZT1 – ZT13 | -1.207 | 0.8291 |
|  |  | ZT1 – ZT17 | -1.843 | 0.4592 |
|  |  | ZT1 – ZT21 | -2.369 | 0.2067 |
|  |  | ZT5 – ZT9 | -1.269 | 0.7985 |
|  |  | ZT5 – ZT13 | -0.214 | 0.9999 |
|  |  | ZT5 – ZT17* | -0.850 | 0.9547 |
|  |  | ZT5 – ZT21 | -1.377 | 0.7400 |
|  |  | ZT9 – ZT13 | 1.054 | 0.8943 |
|  |  | ZT9 – ZT17 | 0.419 | 0.9981 |
|  |  | ZT9 – ZT21 | -0.108 | 1.0000 |
|  |  | ZT13 – ZT17 | -0.635 | 0.9871 |
|  |  | ZT13 – ZT21 | -1.162 | 0.8500 |
|  |  | ZT17 – ZT21 | -0.527 | 0.9945 |

Table S8. Contrasts within each timepoint between ALAN+ and ALAN- mosquitoes for heads of adult females of *Cx. pipiens*. For all GLMs, contrasts are reported with a z ratio and for all LMs contrasts are reported with a t ratio.

| **Gene** | **Timepoint (Zeitgeber)** | **Estimate** | **SE** | **t Ratio**  **or**  **z Ratio** | **p-Value** | **df** |
| --- | --- | --- | --- | --- | --- | --- |
| *cycle* | ZT1 | 0.24026 | 0.0922 | 2.606 | 0.2112 | 48 |
|  | ZT5 | 0.3122 | 0.0922 | 3.386 | **0.0349** | 48 |
|  | ZT9 | 0.41913 | 0.0922 | 4.546 | **0.0011** | 48 |
|  | ZT13 | 0.89924 | 0.0922 | 9.754 | **<0.0001** | 48 |
|  | ZT17 | 0.49158 | 0.0922 | 5.332 | **0.0001** | 48 |
|  | ZT21 | 0.27460 | 0.0922 | 2.978 | 0.0957 | 48 |
| *Clock* | ZT1 | 0.007555 | 0.00323 | 2.342 | 0.3387 | 48 |
|  | ZT5 | 0.008591 | 0.00323 | 2.663 | 0.1887 | 48 |
|  | ZT9 | 0.006111 | 0.00323 | 1.894 | 0.6208 | 48 |
|  | ZT13 | 0.008074 | 0.00323 | 2.503 | 0.2565 | 48 |
|  | ZT17 | -0.005125 | 0.00323 | -1.589 | 0.8060 | 48 |
|  | ZT21 | 0.005851 | 0.00323 | 1.814 | 0.6732 | 48 |
| *period* | ZT1 | 0.7520 | 0.256 | 2.934 | 0.1075 | 45 |
|  | ZT5 | 0.9277 | 0.272 | 3.413 | **0.0336** | 45 |
|  | ZT9 | 0.2952 | 0.256 | 1.152 | 0.9623 | 45 |
|  | ZT13 | -0.1716 | 0.272 | -0.631 | 0.9993 | 45 |
|  | ZT17 | -0.2880 | 0.256 | -1.124 | 0.9674 | 45 |
|  | ZT21 | 0.0535 | 0.272 | 0.197 | 1.0000 | 45 |
| *timeless* | ZT1 | 0.016174 | 0.00999 | 1.619 | 0.7946 | Inf |
|  | ZT5 | 0.029471 | 0.00743 | 3.967 | **0.0024** | Inf |
|  | ZT9 | -0.014931 | 0.02951 | -0.506 | 0.9999 | Inf |
|  | ZT13 | -0.059953 | 0.05900 | -1.016 | 0.9845 | Inf |
|  | ZT17 | -0.047977 | 0.08683 | -0.553 | 0.9998 | Inf |
|  | ZT21 | 0.000729 | 0.02922 | 0.025 | 1.0000 | Inf |
| *Pdp1* | ZT1 | 0.2787 | 0.121 | 2.304 | 0.3602 | 48 |
|  | ZT5 | 0.2841 | 0.121 | 2.349 | 0.3349 | 48 |
|  | ZT9 | 0.1432 | 0.121 | 1.184 | 0.9561 | 48 |
|  | ZT13 | 0.0891 | 0.121 | 0.737 | 0.9979 | 48 |
|  | ZT17 | -0.0926 | 0.121 | -0.765 | 0.9973 | 48 |
|  | ZT21 | 0.0655 | 0.121 | 0.542 | 0.9998 | 48 |
| *cryptochrome 2* | ZT1 | -0.3211 | 0.125 | -2.576 | 0.2238 | 48 |
|  | ZT5 | -0.0868 | 0.125 | -0.696 | 0.9986 | 48 |
|  | ZT9 | 0.1685 | 0.125 | 1.352 | 0.9098 | 48 |
|  | ZT13 | -0.1397 | 0.125 | -1.121 | 0.9681 | 48 |
|  | ZT17 | -0.5373 | 0.125 | -4.310 | **0.0024** | 48 |
|  | ZT21 | -0.2948 | 0.125 | -2.365 | 0.3263 | 48 |

Table S8. Contrasts within each timepoint between ALAN+ and ALAN- mosquitoes for heads of adult females of *Ae. albopictus*. For all GLMs, contrasts are reported with a z ratio and for all LMs contrasts are reported with a t ratio.

| **Gene** | **Timepoint (Zeitgeber)** | **Estimate** | **SE** | **t Ratio**  **or**  **z Ratio** | **p-Value** | **df** |
| --- | --- | --- | --- | --- | --- | --- |
| *cycle* | ZT1 | -0.30230 | 0.204 | -1.479 | 0.8590 | 45 |
|  | ZT5 | -0.57633 | 0.183 | -3.153 | 0.0645 | 45 |
|  | ZT9 | -0.45009 | 0.183 | -2.463 | 0.2772 | 45 |
|  | ZT13 | 0.47511 | 0.194 | 2.451 | 0.2830 | 45 |
|  | ZT17 | 0.19862 | 0.183 | 1.087 | 0.9733 | 45 |
|  | ZT21 | -0.35778 | 0.183 | -1.958 | 0.5794 | 45 |
| *Clock* | ZT1 | 0.0586 | 0.206 | 0.284 | 1.000 | 45 |
|  | ZT5 | -0.4673 | 0.185 | -2.532 | 0.2445 | 45 |
|  | ZT9 | 0.1375 | 0.185 | 0.745 | 0.9977 | 45 |
|  | ZT13 | 0.5721 | 0.196 | 2.293 | 0.1102 | 45 |
|  | ZT17 | 0.1658 | 0.185 | 0.899 | 0.9920 | 45 |
|  | ZT21 | -0.2976 | 0.185 | -1.613 | 0.7928 | 45 |
| *period* | ZT1 | 0.650 | 0.211 | 3.086 | 0.0758 | 45 |
|  | ZT5 | 0.392 | 0.188 | 2.079 | 0.4999 | 45 |
|  | ZT9 | 0.432 | 0.188 | 2.294 | 0.3672 | 45 |
|  | ZT13 | 0.476 | 0.200 | 2.385 | 0.3169 | 45 |
|  | ZT17 | 0.102 | 0.188 | 0.542 | 0.9998 | 45 |
|  | ZT21 | 0.612 | 0.188 | 3.252 | **0.0507** | 45 |
| *timeless* | ZT1 | 0.33318 | 0.138 | 2.433 | 0.2715 | Inf |
|  | ZT5 | -0.38279 | 0.114 | -3.361 | **0.0221** | Inf |
|  | ZT9 | 0.04825 | 0.114 | 0.424 | 1.0000 | Inf |
|  | ZT13 | 0.01682 | 0.121 | 0.139 | 1.0000 | Inf |
|  | ZT17 | 0.07002 | 0.114 | 0.615 | 0.9995 | Inf |
|  | ZT21 | 0.30140 | 0.121 | 2.495 | 0.2342 | Inf |
| *cryptochrome1* | ZT1 | 0.44047 | 0.155 | 2.841 | 0.1326 | 44 |
|  | ZT5 | -0.23280 | 0.139 | -1.1679 | 0.7558 | 44 |
|  | ZT9 | 0.27506 | 0.139 | 1.983 | 0.5626 | 44 |
|  | ZT13 | 0.48771 | 0.147 | 3.315 | **0.0436** | 44 |
|  | ZT17 | 0.64708 | 0.139 | 4.665 | **0.0009** | 44 |
|  | ZT21 | 0.66626 | 0.147 | 4.529 | **0.0014** | 44 |
| *cryptochrome2* | ZT1 | 0.36535 | 0.208 | 1.753 | 0.7111 | 45 |
|  | ZT5 | -0.09640 | 0.186 | -0.517 | 0.9998 | 45 |
|  | ZT9 | 0.15847 | 0.186 | 0.850 | 0.9944 | 45 |
|  | ZT13 | 0.51349 | 0.196 | 2.597 | 0.2164 | 45 |
|  | ZT17 | 0.33884 | 0.186 | 1.818 | 0.6705 | 45 |
|  | ZT21 | -0.17564 | 0.186 | -0.942 | 0.9890 | 45 |

Table S9. Contrasts within each timepoint between ALAN+ and ALAN- mosquitoes for oocytes of *Ae. albopictus*. For all GLMs, contrasts are reported with a z ratio and for all LMs contrasts are reported with a t ratio.

| **Gene** | **Timepoint (Zeitgeber)** | **Estimate** | **SE** | **t Ratio**  **or**  **z Ratio** | **p-Value** | **df** |
| --- | --- | --- | --- | --- | --- | --- |
| *cycle* | ZT1 | -0.0977 | 0.244 | -0.400 | 1.0000 | 48 |
|  | ZT5 | -0.3459 | 0.244 | -1.417 | 0.8858 | 48 |
|  | ZT9 | 0.0857 | 0.244 | 0.351 | 1.0000 | 48 |
|  | ZT13 | -0.0275 | 0.244 | -0.113 | 1.000 | 48 |
|  | ZT17 | 0.3190 | 0.244 | 1.306 | 0.9245 | 48 |
|  | ZT21 | 0.3478 | 0.244 | 1.424 | 0.8827 | 48 |
| *Clock* | ZT1 | 0.09261 | 0.260 | 0.356 | 1.0000 | 45 |
|  | ZT5 | -0.18030 | 0.276 | -0.654 | 0.9991 | 45 |
|  | ZT9 | -0.10363 | 0.300 | -0.345 | 1.0000 | 45 |
|  | ZT13 | 0.40641 | 0.260 | 1.564 | 0.8187 | 45 |
|  | ZT17 | 0.20482 | 0.260 | 0.788 | 0.9967 | 45 |
|  | ZT21 | -0.14312 | 0.260 | -0.551 | 0.9997 | 45 |
| *period* | ZT1 | -0.0967 | 0.206 | -0.470 | 0.9999 | 48 |
|  | ZT5 | -0.0147 | 0.206 | -0.072 | 1.0000 | 48 |
|  | ZT9 | 0.0202 | 0.206 | 0.098 | 1.0000 | 48 |
|  | ZT13 | 0.1694 | 0.206 | 0.822 | 0.9956 | 48 |
|  | ZT17 | 0.1106 | 0.206 | 0.537 | 0.9998 | 48 |
|  | ZT21 | 0.2117 | 0.206 | 1.028 | 0.9811 | 48 |
| *timeless* | ZT1 | -0.000949 | 0.00466 | -0.204 | 1.0000 | 48 |
|  | ZT5 | 0.001783 | 0.00466 | 0.383 | 1.0000 | 48 |
|  | ZT9 | 0.003002 | 0.00466 | 0.644 | 0.9992 | 48 |
|  | ZT13 | -0.002631 | 0.00466 | -0.565 | 0.99997 | 48 |
|  | ZT17 | 0.006197 | 0.00466 | 1.330 | 0.9172 | 48 |
|  | ZT21 | 0.000908 | 0.00466 | 0.195 | 1.0000 | 48 |
| *cryptochrome1* | ZT1 | -0.2089 | 0.263 | -0.794 | 0.9965 | 48 |
|  | ZT5 | 0.2738 | 0.263 | 1.040 | 0.9797 | 48 |
|  | ZT9 | 0.10722 | 0.263 | 0.407 | 1.0000 | 48 |
|  | ZT13 | -0.0174 | 0.263 | -0.066 | 1.0000 | 48 |
|  | ZT17 | -0.0236 | 0.263 | -0.090 | 1.0000 | 48 |
|  | ZT21 | 0.6705 | 0.263 | 2.547 | 0.2362 | 48 |
| *cryptochrome2* | ZT1 | -0.3813 | 0.155 | -2.466 | 0.2740 | 48 |
|  | ZT5 | -0.2982 | 0.155 | -1.929 | 0.5981 | 48 |
|  | ZT9 | -0.1552 | 0.155 | -1.004 | 0.9837 | 48 |
|  | ZT13 | -0.0664 | 0.155 | -0.4290 | 1.0000 | 48 |
|  | ZT17 | 0.0728 | 0.155 | 0.471 | 0.9999 | 48 |
|  | ZT21 | 0.2247 | 0.155 | 1.4540 | 0.8707 | 48 |

Table S10. Summary of Type II Analysis-of-Variance tests on linear models for circadian clock genes in *Cx. pipiens*. All models included time point (ZT), ALAN exposure (ALAN), and their interaction (ZTxALAN).

| **Gene** | **Residual SE**  **or**  **Residual Deviance** | **Model Term** | **Test Statistic** | **DF** | **P-value** |
| --- | --- | --- | --- | --- | --- |
| *cycle* | 0.146  df = 48 | ZT | F = 232.24 | 5 | ***< 0.0001*** |
|  |  | ALAN | F = 136.35 | 1 | ***< 0.0001*** |
|  |  | ZTxALAN | F = 7.00 | 5 | ***< 0.0001*** |
| *Clock* | 0.822  df = 48 | ZT | F = 25.22 | 5 | ***< 0.0001*** |
|  |  | ALAN | F = 15.45 | 1 | ***0.0003*** |
|  |  | ZTxALAN | F = 2.56 | 5 | ***0.039*** |
| *period* | 0.405  df = 45 | ZT | F = 92.01 | 5 | ***< 0.0001*** |
|  |  | ALAN | F = 5.88 | 1 | ***0.019*** |
|  |  | ZTxALAN | F = 3.48 | 5 | ***0.010*** |
| *timeless* | 46.26  df = 48 | ZT | F = 45.76 | 5 | ***< 0.0001*** |
|  |  | ALAN | F = 19.53 | 1 | ***< 0.0001*** |
|  |  | ZTxALAN | F = 1.24 | 5 | 0.307 |
| *Pdp1* | 0.191  df = 48 | ZT | F = 44.18 | 5 | ***< 0.0001*** |
|  |  | ALAN | F = 6.72 | 1 | ***0.013*** |
|  |  | ZTxALAN | F = 1.386 | 5 | 0.246 |
| *cryptochrome2* | 0.018  df = 47 | ZT | F = 206.76 | 5 | ***< 0.0001*** |
|  |  | ALAN | F = 66.45 | 1 | ***< 0.0001*** |
|  |  | ZTxALAN | F = 22.60 | 5 | ***< 0.0001*** |

Table S11. Summary of Type II Analysis-of-Variance tests on linear models for circadian clock genes in maternal *Ae. albopictus*. All models included time point (ZT), ALAN exposure (ALAN), and their interaction (ZTxALAN).

| **Gene** | **Residual SE**  **or**  **Residual Deviance** | **Model Term** | **Test Statistic** | **DF** | **P-value** |
| --- | --- | --- | --- | --- | --- |
| *cycle* | 0.289  df = 45 | ZT | F = 13.17 | 5 | ***< 0.0001*** |
|  |  | ALAN | F = 5.32 | 1 | ***0.026*** |
|  |  | ZTxALAN | F = 4.78 | 5 | ***0.001*** |
| *Clock* | 0.292  df = 45 | ZT | F = 11.12 | 5 | ***< 0.0001*** |
|  |  | ALAN | F = 0.05 | 1 | 0.832 |
|  |  | ZTxALAN | F = 3.79 | 5 | ***0.006*** |
| *period* | 0.298  df = 45 | ZT | F = 30.65 | 5 | ***< 0.0001*** |
|  |  | ALAN | F = 30.51 | 1 | ***< 0.0001*** |
|  |  | ZTxALAN | F = 1.03 | 5 | 0.412 |
| *timeless* | 1.559  df = 43 | ZT | F = 8.13 | 5 | ***< 0.0001*** |
|  |  | ALAN | F = 0.94 | 1 | 0.339 |
|  |  | ZTxALAN | F = 4.61 | 5 | ***0.002*** |
| *cryptochrome1* | 0.219  df = 44 | ZT | F = 9.43 | 5 | ***< 0.0001*** |
|  |  | ALAN | F = 39.84 | 1 | ***<0.0001*** |
|  |  | ZTxALAN | F = 5.65 | 5 | ***0.0004*** |
| *cryptochrome2* | 0.295  df = 45 | ZT | F = 4.58 | 5 | ***0.002*** |
|  |  | ALAN | F = 4.80 | 1 | ***0.034*** |
|  |  | ZTxALAN | F = 2.04 | 5 | 0.091 |

Table S12. Summary of Type II Analysis-of-Variance tests on linear models for circadian clock genes in *Ae. albopictus* oocytes. All models included time point (ZT), ALAN exposure (ALAN), and their interaction (ZTxALAN).

| **Gene** | **Residual SE**  **or**  **Residual Deviance** | **Model Term** | **Test Statistic** | **DF** | **P-value** |
| --- | --- | --- | --- | --- | --- |
| *cycle* | 0.146  df = 48 | ZT | F = 2.09 | 5 | 0.083 |
|  |  | ALAN | F = 0.22 | 1 | 0.640 |
|  |  | ZTxALAN | F = 1.16 | 5 | 0.341 |
| *Clock* | 0.822  df = 48 | ZT | F = 4.40 | 5 | ***0.002*** |
|  |  | ALAN | F = 0.27 | 1 | 0.604 |
|  |  | ZTxALAN | F = 12.93 | 5 | 0.588 |
| *period* | 0.405  df = 45 | ZT | F = 2.38 | 5 | 0.052 |
|  |  | ALAN | F = 0.63 | 1 | 0.431 |
|  |  | ZTxALAN | F = 0.33 | 5 | 0.895 |
| *timeless* | 46.26  df = 48 | ZT | F = 2.14 | 5 | 0.077 |
|  |  | ALAN | F = 0.53 | 1 | 0.470 |
|  |  | ZTxALAN | F = 0.44 | 5 | 0.819 |
| *cryptochrome1* | 0.191  df = 48 | ZT | F = 2.61 | 5 | **0.036** |
|  |  | ALAN | F = 1.55 | 1 | 0.220 |
|  |  | ZTxALAN | F = 1.37 | 5 | 0.254 |
| *cryptochrome2* | 0.018  df = 47 | ZT | F = 1.24 | 5 | 0.304 |
|  |  | ALAN | F = 2.54 | 1 | 0.118 |
|  |  | ZTxALAN | F = 2.16 | 5 | 0.074 |
